# High-resolution association mapping with libraries of immortalized lines from ancestral landraces

**DOI:** 10.1007/s00122-021-03963-3

**Published:** 2021-10-20

**Authors:** Tobias Würschum, Thea M. Weiß, Juliane Renner, H. Friedrich Utz, Alfons Gierl, Rafal Jonczyk, Lilla Römisch-Margl, Wolfgang Schipprack, Chris-Carolin Schön, Tobias A. Schrag, Willmar L. Leiser, Albrecht E. Melchinger

**Affiliations:** 1 Institute of Plant Breeding, Seed Science and Population Genetics, University of Hohenheim, 70599 Stuttgart, Germany; 2 State Plant Breeding Institute, University of Hohenheim, 70599 Stuttgart, Germany; 3 Genetics, Technical University of Munich, Wissenschaftszentrum Weihenstephan, 85354 Freising, Germany; 4 Plant Breeding, TUM School of Life Sciences, Technical University of Munich, 85354 Freising, Germany

## Abstract

**Key message:**

Association mapping with immortalized lines of landraces offers several advantages including a high mapping resolution, as demonstrated here in maize by identifying the causal variants underlying QTL for oil content and the metabolite allantoin.

**Abstract:**

Landraces are traditional varieties of crops that present a valuable yet largely untapped reservoir of genetic variation to meet future challenges of agriculture. Here, we performed association mapping in a panel comprising 358 immortalized maize lines from six European Flint landraces. Linkage disequilibrium decayed much faster in the landraces than in the elite lines included for comparison, permitting a high mapping resolution. We demonstrate this by fine-mapping a quantitative trait locus (QTL) for oil content down to the phenylalanine insertion F469 in *DGAT1-2* as the causal variant. For the metabolite allantoin, related to abiotic stress response, we identified promoter polymorphisms and differential expression of an allantoinase as putative cause of variation. Our results demonstrate the power of this approach to dissect QTL potentially down to the causal variants, toward the utilization of natural or engineered alleles in breeding. Moreover, we provide guidelines for studies using ancestral landraces for crop genetic research and breeding.

**Supplementary Information:**

The online version contains supplementary material available at 10.1007/s00122-021-03963-3.

## Introduction

Landraces of crop species have been the traditional variety type cultivated under extremely diverse environmental conditions for centuries before being replaced by modern elite cultivars of lines and hybrids. Collected from the entire ecogeographic distribution of the species, the large number of landraces stored in gene banks harbor unique genetic diversity not present in elite breeding material (McCouch et al. 2013; Langridge and Waugh 2019). Thus, they represent a rich reservoir for plant breeding to meet the global challenges of agriculture (Dwivedi et al. 2016; Navarro et al. 2017; Mayer et al. 2020). Nevertheless, landraces of most crops remain largely untouched as they are phenotypically and genetically unexplored. Recent progress in efficient development of pure-breeding lines by the doubled-haploid technology (Melchinger et al. 2017; Chaikam et al. 2019; Kalinowska et al. 2019) or speed breeding (Watson et al. 2018; Jähne et al. 2020) opens new possibilities to exploit this treasure for research and breeding.

Association mapping has become a routinely applied tool in plant genetic research (Yu et al. 2006; McMullen et al. 2009; Navarro et al. 2017). Available experimental approaches are based on panels with more or less diversity, each of which has its limitations. If a panel of very diverse genotypes is used, potentially a worldwide collection, the mapping resolution can be high, but population structure with its confounding effects is present and in addition, many traits will be confounded by adaptation issues of the employed material. If, by contrast, only adapted material is used, this often lacks the rapid decay of linkage disequilibrium required for fine-mapping. With recent advances in gene editing, however, the aim is now to go beyond quantitative trait loci (QTL) down to the level of the underlying genes and the causal alleles.

As an alternative approach, association mapping in landraces has previously been suggested, which is best realized with representative samples of immortalized, homozygous lines (Reif et al. 2005; Strigens et al. 2013; Böhm et al. 2017; Mayer et al. 2017; Melchinger et al. 2017). The advantages of association mapping with immortalized libraries of landraces can be summarized as follows: (1) The mapping populations can display a rapid decay of linkage disequilibrium, because like in humans, for allogamous crop species they are derived from panmictic populations with accumulated historical and evolutionary recombination, which offers excellent conditions for high-resolution mapping. Mayer et al. (2020) have recently used three landrace doubled haploid libraries of maize to map a QTL for tillering to a 1.3-Mb interval on chromosome 1, in which the leading haplotype pointed to a 46-kb interval overlapping perfectly with the well-known *teosinte branched 1* locus and its regulatory upstream region. (2) Within mapping populations of immortalized lines from landraces, an absence of population structure is expected. (3) Using immortalized lines for phenotyping in replicated trials warrants high heritability. Moreover, QTL detection power with fully homozygous lines is at least doubled compared with non-inbred populations and provides estimates of additive effects not confounded with dominance effects. (4) The test statistic for QTL detection depends on contrasts between genotypes from the same landrace and, therefore, avoids artifacts due to extreme differences between genotypes for adaptation to the test environments. The recently proposed FOAM design also uses landraces for association mapping by sampling one individual per landrace for genotyping and phenotyping its testcross progeny with a common tester (Navarro et al. 2017). In contrast to mapping within landraces, however, using highly diverse landrace collections faces the same problem as other diverse panels of having confounding effects of adaptation on target traits.

Maize kernel oil is a valuable renewable resource for food, feed, and bioenergy. Thus, manipulating oil content and a tailored design of the oil composition are attractive targets for a sustainable bioeconomy. Purines are essential components of nucleic acids and other cellular components, and in contrast to animals, plants are capable of a complete degradation of the purine ring to recycle both carbon and nitrogen (Zrenner et al. 2006; Werner and Witte 2011). Allantoin is an intermediate metabolite of this purine catabolism and is metabolized to allantoate by the enzyme allantoinase.

The aim of this study was to assess the potential of libraries of immortalized lines from landraces for crop genetic studies. We performed genome-wide association mapping with doubled haploid libraries of ancestral landraces of maize and demonstrate the potentially high mapping resolution down to causal allelic variants for an oil content QTL and a QTL for the metabolite allantoin. In addition, we provide general conclusions and guidelines for the future utilization of libraries of immortalized lines from landraces for research and breeding.

### Material and methods

#### Plant material and phenotyping of agronomic traits

The basis of our study was the plant materials and phenotypic data taken from a field experiment with 460 inbred lines from the maize breeding program of the University of Hohenheim, described by Böhm et al. (2017). Besides 18 lines of miscellaneous origin, the experiment included 53 elite Flint lines (EF) and a total of 389 doubled-haploid (DH) lines developed from six Flint landraces originating from different countries: Campan-Galade (CG, France), Gelber Badischer (GB, Germany), Strenzfelder (SF, Germany), Rheintaler (RT, Switzerland), Walliser (WA, Switzerland), and Satu Mare (SM, Romania). Field trials for evaluating the per se performance of all lines were conducted in four agro-ecologically diverse environments in Germany in 2013, always using a 46 × 10 α-lattice design with two replications and single-row plots. The four agronomic traits analyzed in this study represent different trait categories: the growth trait early vigor (EV), the yield component trait kernel row number (KRN), the disease resistance trait Fusarium ear rot resistance (FUS), and the quality trait oil content (OC), that were assessed as detailed by Böhm et al. (2017), and showed high heritabilities of 0.82, 0.83, 0.71 and 0.91, respectively, calculated as heritabilities among DH lines within landraces and subsequently averaged across the six landraces. Early vigor was assessed on a scale from 1 (no shoot viable) to 9 (excellent shoot vigor). Fusarium ear rot resistance was assessed just before harvest on a scale from 1 (all ears with symptoms) to 9 (all ears showing no symptoms).

#### SNP genotyping and imputation

A molecular characterization of these doubled haploid landrace libraries has been reported by Melchinger et al. (2017). For genotyping, genomic DNA was extracted from pooled leaf tissue samples of about six plants using the CTAB method following standard protocols. All 53 EF lines and 358 DH lines randomly sampled from the six landraces were assayed with the MaizeSNP50 BeadChip from Illumina® (Ganal et al. 2011) containing 56,110 unique SNPs. Additional genotypes with available marker profiles, not part of this study, were included to improve the imputation step and removed afterward; 426 landrace DH lines and 229 EF lines were used for imputation. Imputation was done separately in the landrace DH lines and in the EF lines. Before this step, landrace genotypes with more than 20% missing marker values or more than 5% heterozygous scores were excluded from all further analyses. Markers with more than 50% missing values or more than 5% heterozygous calls in the landrace genotypes were removed from the data set; the remaining heterozygous scores were set to NA. Imputation was carried out with BEAGLE 5.0 (Browning et al. 2016). The final dataset comprised a panel of 358 landrace lines with both genotypic and phenotypic data. The same markers that remained for the landrace DH lines were filtered in the EF lines, and imputation was performed separately within the EF lines. Fifty-one EF lines with genotypic and phenotypic data remained for analysis. For analyses comparing the landraces with the elite lines (e.g., LD, PCoA, MAF), the dataset with 358 landrace DH lines and 51 EF lines was filtered for markers that showed a minor allele frequency (MAF) < 1% in the landrace DHs or in the EF lines, as these were assumed to more likely be genotyping errors, resulting in 39,054 markers. Thus, applying this threshold, markers can be monomorphic in either the landrace DH lines or the EF lines, but in either of the two must have a minor allele frequency > 1%.

In addition, 92 S_0_ plants from five of the six landraces (GB, *N* = 23; RT, *N* = 23; SF, *N* = 23; SM, *N* = 12; WA, *N* = 11) were genotyped with the Affymetrix® Axiom® Maize Genotyping Array (Unterseer et al. 2014), containing 616,201 unique SNPs. The high-density genotypic data of these S_0_ plants were used to increase the marker density in the landrace DH lines of the mapping population by imputation. We filtered these markers for ‘PolyHighResolution’ markers and used only those for further analyses. Imputation was again carried out with BEAGLE 5.0, first within the 92 S_0_ plants, then using them as a reference panel to impute the landrace mapping population. Subsequently, the panel of 358 landrace lines was filtered for markers with a minor allele frequency < 5%, that were removed from the data set, resulting in 281,881 markers. The EF lines were not imputed with the 600 k data from the landrace S_0_ plants in order to avoid imputation of landrace-specific alleles or haplotypes into the elite lines. Thus, the final data set used for association mapping consisted of 358 landrace DH lines (CG, *N* = 19; GB, *N* = 51; RT, *N* = 34; SF, *N* = 55; SM, *N* = 104; WA, *N* = 95) and 22,095 markers from the 50 k array, while a total of 281,881 SNP markers resulting after the imputation from the 600 k data were used to assist the fine-mapping. The chromosomal positions of these SNPs and the candidate genes identified in subsequent analyses refer to the B73 reference genome (B73 RefGen_v4) (Jiao et al. 2017).

#### Metabolite analyses

For the laboratory analysis of metabolites, we randomly sampled five seeds from the same seed lot as used for the field trials from each of the 460 lines. From each seed, 60 mg of coarsely ground material was used for extraction of metabolites and derivatized according to the method of Lisec et al. (2006). Odd-carbon number alkanes were used as retention index standards. GC–MS analysis was carried out on a Pegasus IV GC-TOFMS (Leco, St. Joseph, Michigan, USA) equipped with a multipurpose sampler and a cold injection system (Gerstel, Mülheim, Germany). One microliter samples were injected at 65 °C, and CIS was ramped at 12 °C/s to a final temperature of 250 °C. Gas chromatography was performed by a 10 m guard column and a 30 m × 0.35 mmi.d. Optima 35 MS separation column with a 0.25 μm film at 2 ml/min constant helium flow and an oven program ramping from 85 to 360 °C at 12 °C/min. Mass spectra were acquired by − 70 V EI at 250 °C in the mass range of 50–800 m/z at 50 spectra/s. Derivatized samples were measured within 20 h.

Data preprocessing and peak quantification were performed using the xcms (Smith et al. 2006) and CAMERA (Kuhl et al. 2012) R software packages. Annotation was carried out with the ChromaTOF Software, version 4.5 (Leco, St. Joseph, Michigan, USA), based on the NIST 11 Mass Spectral Library (Scientific Instrumental Services, USA), the FAMEs Fatty Acid Methyl Esters Mass Spectral Database (Mondello 2011, Wiley, UK), the Golm Metabolome Database (Hummel et al. 2010) and an in-house compound library at TU Munich (Römisch-Margl, unpublished data).

We used a resolvable incomplete block design (40 × 12 α-design) for sample preparation and chemical analysis of the five biological replicates of the 460 genotypes. Days on which the analysis was carried out were treated as incomplete blocks. Twelve consecutive days constituted a complete replicate so that the entire design required 60 working days. While the initial design was for 480 genotypes, a number divisible by 40, which was the maximum number of samples processable per day, we defined a group of 20 dummy genotypes that were later dropped in the final design so that the incomplete blocks (corresponding to days) comprised either 38 or 39 genotypes.

Out of the 288 metabolites measured by this assay, we exemplarily chose allantoin, stigmastan, galacturonic acid and an unknown metabolite UN_322_870, as these all showed high heritabilities of 0.87, 0.68, 0.91 and 0.91, respectively. Three of the metabolites were chosen to have known identity (allantoin, stigmastan, and galacturonic acid) and one to have an unknown identity. In addition to their high heritabilities, all four metabolites produced clear peaks in the Manhattan plots, indicating at least one QTL with large enough effect to warrant fine-mapping.

#### Phenotypic analyses

For the agronomic data, Best Linear Unbiased Estimates (BLUEs) for each genotype were obtained by the two-step analysis detailed by Böhm et al. (2017). First, adjusted-entry means were calculated with ordinary lattice analysis for data from each environment that in a second step were used to estimate BLUEs in a combined analysis across all environments (Cochran and Cox, 1957).

Following Riedelsheimer et al. (2012), raw data of the metabolites were first subjected to a Box–Cox power transformation to meet approximately the assumptions of a Gaussian normal distribution (Box and Cox, 1964). For each metabolite, an ordinary lattice analysis of variance for the incomplete block design was conducted with covariance adjustment for the position of the sample analyzed within an incomplete block (day). Outlier detection was performed by examining residuals standardized by the median absolute deviation (MAD), known to yield robust estimates (Leys et al. 2013). Subsequently, one percent of the most extreme data points identified by this procedure were treated as missing values, including 23 seeds for which concentrations of several metabolites were suspiciously high. This procedure was repeated a second time, and afterward BLUEs for each genotype and metabolite were calculated. Heritabilties (${h}^{2}$) of the metabolites were estimated after Piepho and Möhring (2007). All mixed model analyses were performed with the R package ‘ASReml’ (Butler et al. 2009).

#### Genetic analyses

All analyses described in this section were based on the 39,054 markers from the MaizeSNP50 array described above. Minor allele frequency was assessed in the 358 DH lines and in the 51 EF lines, as the frequency of the allele that in the 358 landrace DH lines is the minor allele. Notably, this allele may be the major allele in the 51 EF lines. To evaluate variations in MAF along chromosomes, a sliding window was applied with 5 Mbp in each direction from the chosen position and a stepsize of 1 Mbp. For each window the mean MAF was calculated. To visualize differences in MAF between the landraces and the elite lines, we calculated ∆ MAF as the MAF in the 358 landrace DHs or each landrace separately minus the MAF in the 51 EF lines. Thus, a positive ∆ MAF illustrates a higher frequency of this allele in the landraces, whereas a negative value shows a higher frequency in the elite lines.

Population structure among the 358 DH lines from the six landraces and the 51 EF lines was investigated by principal coordinate analysis (PCoA) based on the genomic kinship calculated by Method 1 of VanRaden (2008). The neighbor joining tree was calculated with R package ‘ape’ (Paradis et al. 2004).

Linkage disequilibrium (LD) was calculated as the squared correlation (${r}^{2}$) between pairs of markers, separately in each of the landraces, in all 358 landrace DH lines and in the 51 EF lines (Hill and Robertson 1966). The decay of LD with physical distance between markers was assessed by fitting a cubic smoothing spline to the data. To assess LD along chromosomes, the same sliding window as for the MAF was applied. For each window, the mean LD was calculated and the distance after which LD decayed below the threshold of ${r}^{2}$ = 0.2 was determined based on the fitted cubic smoothing spline. For calculating the linkage phase similarity (LPS) in different populations, the cosine similarity was used as described by Schopp et al. (2017). LPS was assessed for a distance of 1 Mbp within bins of 20 kb. For the LPS within landraces and within the elite Flint lines, two subsets of equal size were randomly sampled from each group, for the landraces taking half of the lines from each landrace in each subset, and the results averaged over 100 replications.

#### Association mapping

Genome-wide association mapping was performed in the mapping panel of 358 landrace DH lines, based on the BLUEs of the four agronomic traits and four metabolites, and the 281,881 markers obtained in this panel after imputation and quality checks. A linear mixed model was used for association mapping, incorporating a kinship matrix calculated based on the genome-wide marker data to correct for relatedness (Yu et al. 2006). To account for inter-population differences, a fixed effect was additionally included in the model that defines the origin of each DH line as one of the six landraces. Genome-wide association mapping was done with the R package ‘GenABEL’ (Aulchenko et al. 2007). As significance threshold, we applied a conservative Bonferroni-corrected threshold of *P* < 0.05 at the genome-wide level and show both the threshold for markers from the 50 k array only, as well as for all markers resulting after the imputation. The results from the genome-wide scan for marker–trait associations were visualized in Manhattan plots, drawn as circular plots with ‘Circos’ (Krzywinski et al. 2009). To identify candidate genes underlying the identified peaks in the Manhattan plots, we used the genome browser at maizeGDB (www.maizegdb.org/gbrowse) to zoom into the respective chromosomal regions.

#### Evaluation of candidate genes

For oil content, we sequenced *DGAT1-2* (gene symbols: Zm00001d036982, GRMZM2G169089, LOC103629820) and for allantoin the allantoinase (gene symbols: Zm00001d026635, GRMZM2G173413, LOC100274212). Longer genomic regions were PCR-amplified as shorter, overlapping products. The primers used for (1) the PCRs, (2) the sequencing of the PCR products, (3) the KASP markers developed for some of the identified polymorphisms, and (4) the markers for the allantoinase qPCR are provided in Supplementary Table 1.

The proportion of genotypic variance (${\pi }_{G}$) explained by these polymorphisms was estimated by fitting them in a linear model to obtain ${R}_{\mathrm{adj}}^{2}$, from which the proportion of explained genotypic variance was derived as the ratio ${\pi }_{G}$ = ${R}_{\mathrm{adj}}^{2}$/${h}^{2}$, where ${h}^{2}$ is the heritability of the trait (Utz et al. 2000).

Analysis of several polymorphisms in the allantoinase gene and its promoter suggested the promoter polymorphisms to be causal. In that case the polymorphisms might result in differential gene expression. To study the expression of the allantoinase, we first formed a discovery set of lines comprising 16 genotypes. From each landrace, one line with the insertion and one line with the deletion in the promoter at position −350 were selected. For the elite lines, the only line with insertion and three lines with the deletion were chosen. These lines were grown in a randomized design in a growth chamber, and shoot and root tissue of seedlings were harvested. RNA of two biological replicates was extracted with the Qiagen RNeasy® Plant Kit and reverse transcribed into cDNA with the M-MuLV reverse transcriptase from Genaxxon bioscience. Gene-specific primers were designed for the allantoinase, and elongation factor 1 alpha (*EF1α*) was used as control gene (Lin et al. 2014) (Table S1). Quantitative PCRs were performed on a Roche LightCycler® 480 II with the Genaxxon bioscience GreenMasterMix and four technical replications per biological replicate, resulting in eight expression values per genotype. To analyze allantoinase expression in the landrace ‘Walliser,’ ten lines with the insertion and ten lines with the deletion were selected to sample shoot and root tissue of seedlings from a field trial. The trial was grown at the experimental station Heidfeldhof of the University of Hohenheim in Stuttgart, Germany, in 2020, with two replications. Plants were sampled from both field replications and RNA isolated twice from each sample. With each cDNA sample, 2 to 3 qPCR runs were performed, resulting in 4 to 6 expression values per biological sample and, across the two field replications, in 8 to 12 expression values per genotype.

### Results

#### Maize landraces present an untapped reservoir of variation

**Fig. 1:**
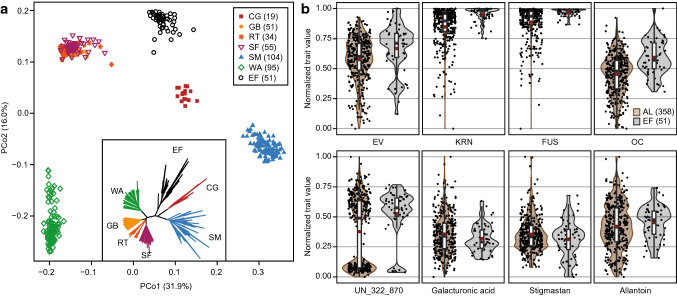
Molecular diversity and phenotypic variation of doubled haploid libraries of six maize ancestral landraces. **a** Principal coordinate analysis and neighbor-joining tree based on genome-wide marker data for doubled haploid lines from six ancestral landraces and elite Flint lines (EF). The number of lines per group is given in parentheses; percentages in parentheses refer to the proportion of genotypic variance explained by the first and second principal coordinate. **b** Phenotypic variation of four agronomic traits (*EV* early vigor; *KRN* kernel row number; *FUS* Fusarium resistance; *OC* oil content) and four metabolites shown as normalized trait values for the 358 ancestral landrace (AL) doubled haploid lines and 51 elite Flint lines

Our population comprised 358 immortalized maize lines from six geographically diverse ancestral European Flint landraces that were developed by the doubled-haploid technology, as well as 51 elite Flint inbred lines included for comparison. Molecular differentiation of the landraces and the elite lines was supported by the results of the principal coordinate, phylogenetic, and minor allele frequency analyses (Fig. 1a, Fig. S1, S2). Clustering of the landraces was in agreement with their geographic origin, with ‘Satu Mare’ from Romania being most distant from the other landraces, which originated from the Alp/Pyrenean region (‘Walliser,’ ‘Campan-Galade’) and Upper Rhine valley (‘Gelber Badischer,’ ‘Rheintaler’). ‘Strenzfelder’ was presumably derived from ‘Gelber Badischer,’ explaining their close association.

All traits showed a high heritability and a broad phenotypic range, with the variation present in the doubled haploid libraries of landraces often exceeding that of the elite lines (Fig. 1b, Fig. S3). For susceptibility to diseases or other undesired characteristics, the variation was expectedly lower in the elite material, because breeders have strongly selected against these traits since decades. Thus, the allele frequencies and consequently the power of QTL detection for such traits should be higher in landraces.

#### Rapid decay of linkage disequilibrium in maize landraces

**Fig. 2:**
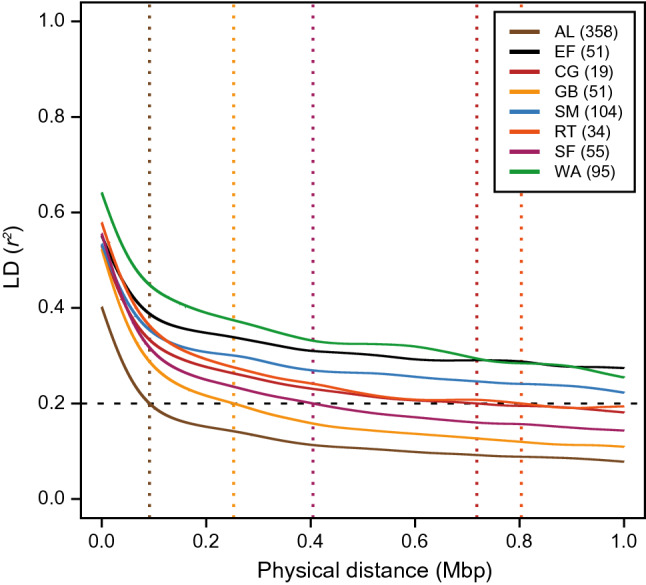
Rapid decay of linkage disequilibrium in ancestral landraces. Linkage disequilibrium (LD) decay with physical distance shown for the ancestral landraces (AL), the elite Flint (EF) lines, and for each landrace separately. The distance after which LD decays below the threshold of 0.2 (dashed horizontal line) is indicated by the dotted vertical lines

The crucial factor determining the mapping resolution of association mapping is the decay of linkage disequilibrium with distance from the causal allelic variants. Linkage disequilibrium decayed much faster in the landrace association mapping population (*r*^*2*^ ≤ 0.2 at 0.18 Mbp) than in the elite lines (*r*^*2*^ ≤ 0.2 at 3.66 Mbp) (Fig. 2). Within each landrace, linkage disequilibrium also decayed faster compared to the elite lines, but with substantial variation among them (*r*^*2*^ ≤ 0.2 ranging from 0.34 to 2.93 Mbp), which is in line with previous findings (Mayer et al. 2017). Analysis of linkage disequilibrium along chromosomes revealed the expected slower decay in centromeric regions, but substantiated the much faster decay in non-centromeric regions in the landraces, facilitating a higher mapping resolution in most of the genome (Fig. S4). This difference between the landraces and elite lines was further corroborated by the short persistence of the same linkage phase (Fig. S5). Collectively, these results emphasize the importance of analyzing linkage disequilibrium before selecting landraces as targets for association mapping, and highlight the potential of the present panel for high-resolution mapping.

#### Landraces enable high-resolution mapping of causal variants

**Fig. 3:**
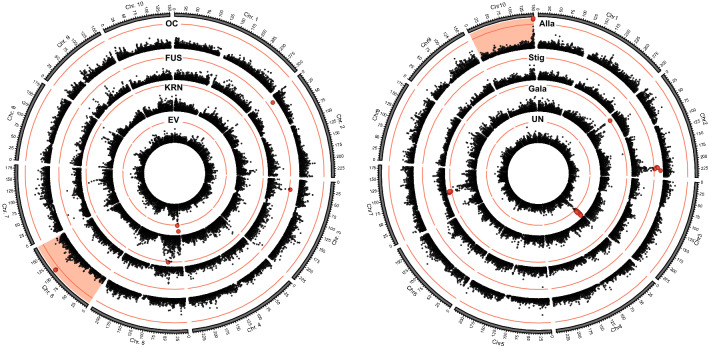
Genome-wide results of association mapping in the landrace panel. Manhattan plots for the results from association mapping in the 358 doubled haploid lines from landraces shown for four agronomic traits (*OC* oil content; *FUS* Fusarium resistance; *KRN* kernel row number; *EV* early vigor) and four metabolites (Alla, allantoin; Stig, stigmastan; Gala, galacturonic acid; UN, UN_322_870). The red lines indicate the significance threshold (Bonferroni corrected *P* < 0.05), significant associations are shown in red. Chromosomes used for further fine-mapping are highlighted red

Genome-wide association mapping in the panel of 358 immortalized landrace lines yielded associations for all traits (Fig. 3, Table S2). We chose two of these QTL with clear signal in the Manhattan plot, a QTL of the agronomic trait oil content and a QTL for the metabolite allantoin, to explore the mapping resolution permitted by this approach.

**Fig. 4:**
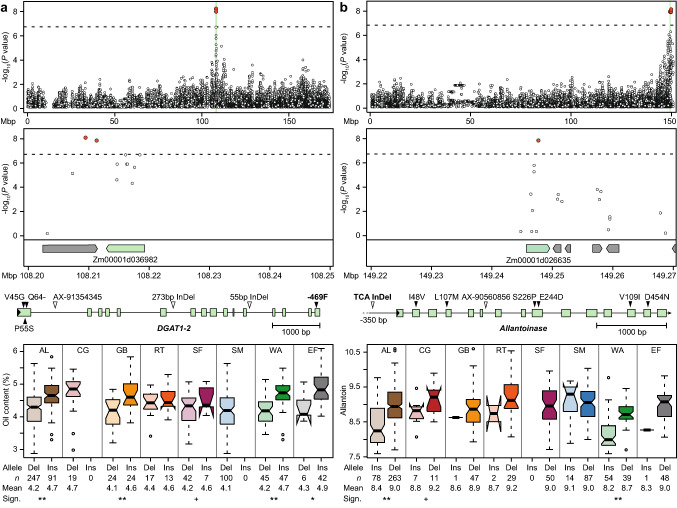
Identification of candidate genes and potential causal variants. **a** Fine-mapping of the oil content QTL on chromosome 6 and identification of *DGAT1-2* (Zm00001d036982, highlighted in green) with the causal phenylalanine insertion at position 469. The dashed horizontal line indicates the significance threshold (Bonferroni corrected *P* < 0.05), significant associations are shown in red. The boxplots show the effect of this insertion/deletion (Ins/Del) in the ancestral landraces (AL), each of the landraces, and elite Flint lines (EF). + , *, **, significant at *P* < 0.1, 0.05, 0.01, respectively. **b** Fine-mapping of the QTL for allantoin content on chromosome 10 and identification of potentially causal polymorphisms in the allantoinase gene (Zm00001d026635) and promoter. The boxplots show the effect of the TCA insertion/deletion in the promoter region at position -350

We fine-mapped the major QTL for oil content on chromosome 6 to a narrow region containing *DGAT1-2*, a gene encoding an acyl-CoA:diacylglycerol acyltransferase that catalyzes the final step of oil biosynthesis (Fig. 4a). *DGAT1-2* is known to have a comparably large effect on oil content (Li et al. 2013); in particular, a phenylalanine insertion at position 469 (F469) was shown to be the causal polymorphism for increased oil and oleic acid concentration (Zheng et al. 2008). Sequencing of *DGAT1-2* identified the polymorphisms described previously, including the causal phenylalanine insertion (Table S3).

For the metabolite allantoin, a major association signal was identified on chromosome 10. Fine-mapping identified the allantoinase as a strong candidate for this QTL, and sequencing revealed six non-synonymous polymorphisms in the coding region as well as several polymorphisms in the promoter region (Fig. 4b, Fig. S6-7, Tables S4-7). The two landrace populations that are likely most informative and may have driven the identification of this QTL are ‘Campan Galade’ and ‘Walliser,’ because the marker from the SNP array (AX-90560856) segregates with almost equal allele frequencies and lines with the different marker alleles show a clear difference in allantoin content. For these two landraces, the non-synonymous mutations L107M and D454N are polymorphic. However, KASP markers for L107M and D454N were not significantly associated with the trait and explained almost nothing of the genotypic variance (Table S7). By contrast, the promoter polymorphisms matched the pattern of the SNP marker in the subset of sequenced lines. Moreover, the KASP marker tagging the TCA InDel at position -350, developed as a proxy for the various promoter polymorphisms showing the same pattern, explained the highest proportion of genotypic variance in the entire panel. This suggested that one or several polymorphisms in the promoter region or in another regulatory region being in LD with it, contribute to this QTL.

**Fig. 5:**
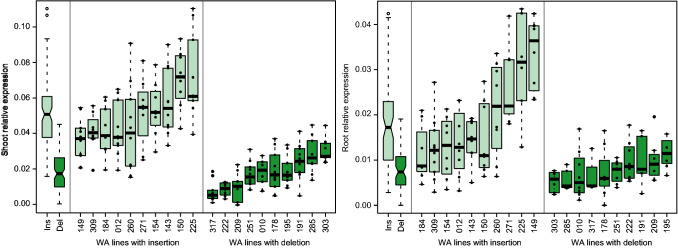
Differential expression of the *Allantoinase*. *Allantoinase* mRNA expression in shoots and roots of 20 field-grown lines from the landrace ‘Walliser’ (WA) with either the insertion or deletion at the promoter position − 350. Ins and Del show the relative expression of all lines carrying the insertion or deletion, respectively

Particularly for the landrace ‘Walliser,’ both promoter variants are present and showed significant (*P* = 4.0e−10) differences in allantoin content (Table S8). We consequently assessed transcriptional expression of the allantoinase in shoots and roots of maize seedlings grown under controlled conditions, which for ‘Walliser’ indicated differential expression (Fig. S8). Subsequent quantitative PCR of 20 field-grown lines of this landrace confirmed significant differences in *Allantoinase* mRNA levels between the two promoter variants in both shoots (*P* = 1.0e−26) and roots (*P* = 2.3e−10) (Fig. 5). The lines with the insertion had an on average 2.8-fold higher expression level in shoots and a 2.3-fold higher expression level in roots. Lines with the allele characterized by a higher allantoin content had a lower *Allantoinase* expression, which is in line with a reduced turnover of allantoin in this metabolic pathway.

## Discussion

### Improvement of oil content and allantoin content in maize

In this study, we exemplarily chose QTL for oil content and allantoin content, as both traits produced a clearly defined peak in the Manhattan plot that warranted fine-mapping. For oil content, this identified the well-known phenylalanine insertion at position F469 in *DGAT1-2* (Zheng et al. 2008). The allele with F469 was found in various maize wild relatives, suggesting it to be ancestral, whereas the 3-bp deletion without F469, carried by inbred lines such as B73 and Mo17, is a more recent mutation selected by domestication or breeding (Zheng et al. 2008). Our finding that the low-oil allele without F469 is present in several European maize landraces corroborates the conclusion that it has arisen during domestication (Chai et al. 2012). While a considerable increase in oil content has been achieved in maize, release of commercial high-oil hybrids has been hampered by its negative association with grain yield and other agronomic traits. Our results corroborate *DGAT1-2* as a target to uncouple oil content and quality from other traits by utilizing natural or engineered alleles in breeding of maize and other crops (Liu et al. 2020).

For allantoin content, the enzyme allantoinase was identified as candidate gene. Further analyses suggested promoter polymorphisms and differential expression of the gene as a possible cause of variation in allantoin content. Notably, this does not rule out an effect of the non-synonymous polymorphisms in the coding region. In fact, different polymorphisms may contribute to the differences in allantoin levels in the different landraces. For example, neither the marker AX-90560856 nor the promoter InDel at position -350 were polymorphic between the two sequenced lines of the landrace ‘Strenzfelder,’ but the non-synonymous mutation D454N was, which in this landrace explained 26.0% of the genotypic variation. In addition to its role in nitrogen recycling, allantoin plays a role in abiotic stress response. Particularly, it was found to accumulate under drought in various plant species (Silvente et al. 2012; Degenkolbe et al. 2013; Casartelli et al. 2019; Khan et al. 2019), but also under high salt (Wang et al. 2016), cold (Kaplan et al. 2004), and sulfate starvation conditions (Nikiforova et al. 2005), with generally higher levels in more stress tolerant genotypes. Our results thus lay the foundation for a targeted exploitation of the allelic diversity controlling allantoin content toward the development of crops with improved stress tolerance and nitrogen-use efficiency.

### Association mapping with landraces

While association mapping in diversity panels or in elite breeding material and related approaches like NAM, MAGIC or the recently proposed FOAM, have all proven their value, association mapping in ancestral landraces has several advantages and thus complements these approaches. Our results demonstrate the high mapping resolution enabled by this approach, despite the rather moderate population size used here. Thus, if larger population sizes as used in the maize NAM and FOAM populations (McMullen et al. 2009; Navarro et al. 2017) are employed, association mapping in ancestral landraces can be expected to be even more powerful. Our results together with the findings of Mayer et al. (2020) give clear evidence that the necessary detection power and high mapping resolution can be reached to dissect the genetic architecture of oligo- or even polygenic traits down to the level of the causal variants. Nevertheless, as for any other mapping approach, the target QTL obviously need to have a sufficiently large effect size to warrant their fine-mapping and potential identification of the underlying genes. Following their validation and further characterization, the identified genes can be utilized for breeding by allele mining in gene banks or by targeted engineering of alleles through gene editing. In addition, high-resolution mapping could enlarge our knowledge on the numerous metabolites with unknown function (Tohge and Fernie 2010) through identification of candidate genes affecting their metabolic pathway, but this warrants further research.

### How to identify the target landraces

**Fig. 6:**
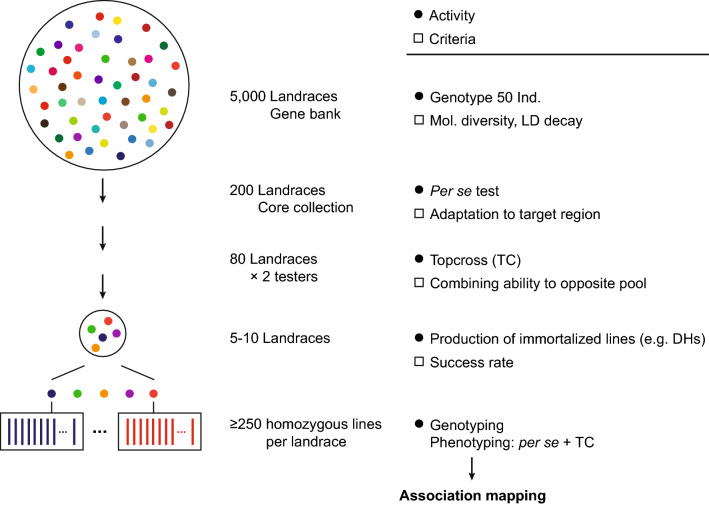
Schematic roadmap for association mapping in landraces. Numbers are given exemplarily for maize. Figure adapted from Mayer et al. (2020)

In view of the large number of landrace accessions stored in gene banks and the immense efforts and expenses for production of immortalized lines from these populations (Melchinger et al. 2017), a multi-stage procedure for choice of the landrace(s) used for association mapping appears best suited (Fig. 6) (Mayer et al. 2020). First, landraces harboring the desired characteristics are required. These can either be collected by a targeted search in environments that suggest the presence of variation for the target trait(s). Alternatively, a core collection of landraces is identified based on passport data and/or molecular information, and subsequently evaluated for the trait(s) of primary interest. These landraces should ideally be well adapted to the environments used for phenotyping to avoid artifacts arising from poor adaptation, which could affect plant development and in turn the target trait(s). Promising landraces selected after this step must be evaluated for their success rate in production of libraries of immortalized lines, as a minimum sample size is required to provide the power to identify causal variants. For maize landraces, for example, there is substantial variation regarding the production of doubled haploid lines, with some landraces being rather recalcitrant (Melchinger et al. 2017). At present, doubled haploid lines can be routinely produced in maize and few other crops. As an alternative, recombinant inbred lines from landraces can be developed by single seed descent, which can be accelerated by applying novel approaches for speed breeding (Watson et al. 2018; Jähne et al. 2020). In addition, the candidate landraces should be fingerprinted with molecular markers to identify those landraces with a rapid linkage disequilibrium decay that facilitates fine-mapping. Analyzing 24 individual S_0_ plants from each landrace with a 5 K SNP array was sufficient for obtaining representative estimates in maize (Mayer et al. 2017).

### How to design the mapping population

An important question is the number of immortalized lines that should be produced for the selected landrace(s). This depends on the available resources as well as on the research objectives. An important consideration is also that most of the molecular and genetic variation rests within landraces and less than one quarter between landraces (Böhm et al. 2017; Goodman et al. 2014). In general, the overall number is limited by the resources required to produce them as well as by the capacity to phenotype them for the target trait(s). Thus, the available resources can be focused on one landrace or be used to establish libraries of several landraces. Choosing a single landrace might be recommended if the focus is on a specific target trait that is rare to find and maybe only present in a particular landrace, or if a landrace is known to show a much stronger variation for it than all other landraces. If several landraces show variation for the target trait(s), the more promising option might be to establish libraries for several of them. Even with a similar variation, the genetic architecture of the target trait may differ and thus, assessing several landraces increases the chance of identifying large-effect QTL suitable for introgression into elite material. If the genetic architecture is identical between landraces, there is no difference whether one or several are chosen. In association mapping, the QTL detection power depends on the size of the mapping population. In our study, the QTL for allantoin content may only segregate in the landrace ‘Walliser’ with 95 individuals, indicating that a population size of around 100 can be sufficient to detect large-effect QTL. Notably, this will also depend on the linkage disequilibrium structure in the QTL region. Thus, a sample size of 250 as used by Mayer et al. (2020) should generally be sufficient to identify large- and medium-effect QTL and only if the aim is to identify also QTL with smaller effect should the sample size be increased further. If the aim is to use the mapping population for different target traits, even some not yet defined when screening the landraces, the chances for success are increased when libraries of several landraces are established. The sample size per landrace library must then be balanced with the number of landraces, potentially with a stronger focus on some landraces that appear most promising. The use of multiple immortalized landraces increases the number of polymorphic QTL and also opens the possibility to test for heterogeneity of QTL effects in different landraces, as frequently observed in QTL studies with multiple bi-parental populations (e.g., Blanc et al. 2006; Liu et al. 2011).

### Maximizing the mapping resolution

The basis for a high mapping resolution in association mapping is a rapid decay of linkage disequilibrium. As expected, linkage disequilibrium was found to decay faster in the landrace population than in the elite lines, which is in line with previous findings (van Inghelandt et al. 2011; Truntzler et al. 2012; Unterseer et al. 2014; Mayer et al. 2017; Brauner et al. 2018). In very diverse collections of maize, the linkage disequilibrium decay can be even faster than in the landraces, but as mentioned, these panels usually have the issue of a strong population structure and non-adaptation to the test environments (Yan et al. 2009).

Maximizing the diversity within each landrace library supports achieving a high mapping resolution. To exclude closely related lines, which may occur if several immortalized lines are recovered from the same S_0_ plant, we recommend to produce an excess of immortalized lines and, based on marker data, choose less related ones for the association mapping panel. In addition, the rapid decay of linkage disequilibrium in landraces must be matched with a high marker density to take full advantage of the high mapping resolution achievable with this approach. The marker density provided by the 50 k SNP array in our study was at the lower end. In view of the advances in sequencing technology and the decreasing costs, whole-genome sequencing should be the method of choice for association mapping in landrace libraries.

### Landrace association mapping and genetic load

Performing association mapping with immortalized lines from landraces instead of the landraces themselves increases the QTL detection power and might have the advantage that detrimental QTL alleles contributing to the genetic load are largely purged from the landraces during the process of producing the immortalized lines. However, in maize a comparison of SNP allele frequencies in the original landrace and the immortalized lines derived from them provided little evidence that this occurs frequently (Melchinger et al. 2017; Zeitler et al. 2020). Nevertheless, detection of QTL with detrimental effects could help to identify different variants of beneficial alleles in elite germplasm and eradicate the negative alleles if still present at low frequencies.

### Joint utilization for hybrid breeding

If the immortalized landrace lines are to be used for broadening the genetic basis in hybrid breeding programs, molecular information about the landraces and elite germplasm can help choosing landraces that maximize the genetic distance to the heterotic group targeted for introgression and the opposite group in the heterotic pattern. This should increase the chances to detect novel alleles absent in breeding populations and improve the heterotic response in hybrids. With this goal in mind, we recommend to additionally evaluate their testcross performance with suitable testers as practiced in choosing the landraces for this study (Böhm et al. 2014).

## Conclusions

The approach of utilizing landraces for association mapping is most suitable for allogamous or partially allogamous crops. For genetically narrow landraces of autogamous crops, it is sufficient to sample a single representative line to be included in a diversity panel, as recently demonstrated for barley (Milner et al. 2019). However, autogamous crops also display a certain degree of outcrossing, and if ancestral landraces have a sufficiently large effective population size and segregate for the trait(s) of interest, association mapping may also be successfully applied to landraces of autogamous crops, which warrants further research.

Association mapping in landraces has special appeal due to the rapid decay of linkage disequilibrium over a short distance and as these may harbor rare alleles absent in elite material. Both criteria are a direct function of the effective population size *N*_*e*_, which depends on both, the evolutionary history of the landraces as well as their collection and maintenance. While the latter, including the strategy for their collection, cannot be changed for gene bank material, for their future use in research and breeding, it is of utmost importance that maintenance of these accessions preserves a large *N*_*e*_, which requires a revision of the number of genotypes and mating schemes currently used by many gene banks.

In conclusion, using libraries of ancestral landraces for association mapping allows tapping a huge, largely unexploited reservoir of genetic resources for genetic research and breeding.

## Electronic supplementary material

Below is the link to the electronic supplementary material.

Supplementary file1 (PDF 1695 KB)

## Data Availability

The phenotypic and genotypic data that supported the findings of this study are available as Supplement (Supplementary Data 1).
