# Supplementary material for: High-resolution association mapping with libraries of immortalized lines from ancestral landraces
Source: Theor Appl Genet. 2021 Oct 20;135(1):243–56. doi: 10.1007/s00122-021-03963-3 (PMC8741726; doi:10.1007/s00122-021-03963-3)
Supplement: Supplementary file 1 — Supplementary file1 (PDF 1695 KB) [file 122_2021_3963_MOESM1_ESM.pdf]

High-resolution association mapping with libraries of immortalized lines from ancestral landraces

Tobias Würschum[1], Thea Mi Weiß[1,2], Juliane Renner[1], H. Friedrich Utz[1], Alfons Gierl[3], Rafal Jonczyk[3], Lilla Römisch-Margl[3], Wolfgang Schipprack[1], Chris-Carolin Schön[4], Tobias A. Schrag[1], Willmar L. Leiser[2], Albrecht E. Melchinger[1]

[1] Institute of Plant Breeding, Seed Science and Population Genetics, University of Hohenheim, 70599 Stuttgart, Germany.

[2] State Plant Breeding Institute, University of Hohenheim, 70599 Stuttgart, Germany.

[3] Genetics, Wissenschaftszentrum Weihenstephan, Technical University of Munich, 85354 Freising, Germany.

[4] Plant Breeding, TUM School of Life Sciences, Technical University of Munich, 85354 Freising, Germany.

✉ Tobias Würschum, email: tobias.wuerschum@uni-hohenheim.de and Albrecht E. Melchinger, email: melchinger@uni-hohenheim.de

ORCID IDs: 0000-0002-7397-7731 (T.W.), 0000-0002-9809-6340 (T.M.W.), 0000-0003-2331-7054 (A.G.), 0000-0001-7025-0855 (R.J.), 0000-0002-9937-5296 (L.R.M.), 0000-0001-5964-5610 (W.L.L), 0000-0002-8346-7786 (A.E.M.)

Supplementary Material

Table S1 Primers used for candidate gene analysis. Primers used to amplify and sequence the *DGAT1-2* and allantoinase candidate genes, primers of the KASP markers developed for some of the identified polymorphisms, and primers used for allantoinase qPCR.

Primer	Sequence (5' – 3')	PCR info	Use
DGAT1-2 sequencing			
DGAT_P1_F2	CCCATACAAACAATGTATGGACCG	Phusion Hot Start, 63.0°C + Touchdown 68-60°C, fragment length 3,304bp	Amplification of part 1 of *DGAT1-2*
DGAT_P1_R2	TTTATCATCCAAATAAGCACTCCTACC		
DGAT_P1_S1	AAGAGGGAGATTGAAAGGGA		Sequencing primer part 1
DGAT_P1_S2	GGTTCACGATAGACTCCGTG		Sequencing primer part 1
DGAT_P1_S3	TTGTACGAGGCACGTTTACT		Sequencing primer part 1
DGAT_P1_S4	ATGCCTGCCGCCTCCGATCG		Sequencing primer part 1
DGAT_P1_S5	TTGTGGGCCTTGGGCTTC		Sequencing primer part 1
DGAT_P2_F2	CTTGTTGAGGACCATATAACTGTGC	Phusion Hot Start II, 63.0°C + Touchdown 68-60°C, fragment length 3,294bp	Amplification of part 2 of *DGAT1-2*
DGAT_P2_R2	CTGTTGAACTGAAGCAAGATACTAACC		
DGAT_P2_S1	GCATTGTTGTTCTGATCGCAG		Sequencing primer part 2
DGAT_P2_S2	GTGTTGAGGATTGAATTAGATGT		Sequencing primer part 2
DGAT_P2_S3	ATGTTCATTTATCTCTAGTAGAAGAA		Sequencing primer part 2
DGAT_P2_S4	CAGGTGTGACTCAGCAGTAC		Sequencing primer part 2
DGAT_P2_S5	GGTCCACCTAACCCTGTTAT		Sequencing primer part 2
DGAT_P3_F2	TGAGCAAGTGAGCCTCCTATATTCC	Phusion Hot Start II, 63.0°C + Touchdown 68-60°C, fragment length 3,200bp	Amplification of part 3 of *DGAT1-2*
DGAT_P3_R2	GGGAAGCTTTTGAGTAACCTTTGTTC		

Primer	Sequence (5’ – 3’)	PCR info	Use
DGAT_P3_S1	CTCATGTACTTAAATGTGATGG		Sequencing primer part 3
DGAT_P3_S2	TGTCATAATAATAATGAATACAACTGA		Sequencing primer part 3
DGAT_P3_S3	GAATTCCACTCAGCACATTAC		Sequencing primer part 3
DGAT_P3_S4	GAACGACTTGTAATTATCCCTGC		Sequencing primer part 3
DGAT_P3_S5	AATGCTTCTATTCCAGGC		Sequencing primer part 3
Allantoinase sequencing			
Alla_P1_F2	CCTTCCGATCGACTAATGGG	Phusion Hot Start II, 63.0°C + Touchdown 68-60°C, fragment length 3,547bp	Amplification of part 1 of allantoinase
Alla_P1_R2	GTTGGCACTACTGCTTGCACTTAC		
Alla_P1_S1	TGGTTGGTCTATGTTTGTG		Sequencing primer part 1
Alla_P1_S1n	GATCTGTTTGGTTGGTCTATG		Sequencing primer part 1
Alla_P1_S2	CTCTGCATAGAGCGTAGAG		Sequencing primer part 1
Alla_P1_S3	AGTAGACGGGCAGGACAG		Sequencing primer part 1
Alla_P1_S4	TTGGTGCCTGCAGTAGAG		Sequencing primer part 1
Alla_P1_S5	GCAGAGCAAATAACAAGC		Sequencing primer part 1
Alla_P1_S6	CCAGAGAATGCCTTCAAC		Sequencing primer part 1
Alla_P2_F2	CTTGTTGAGGACCATATAACTGT	Phusion Hot Start, 63.0°C + Touchdown 68-60°C, fragment length 3,500bp	Amplification of part 1 of allantoinase
Alla_P2_R2	CTGTTGAACTGAAGCAAGATACT		
Alla_P2_S1	GTATAGTTATGGCGGTGG		Sequencing primer part 2
Alla_P2_S2	ATCAGATTTGCATGTCCAC		Sequencing primer part 2

Primer	Sequence (5’ – 3’)	PCR info	Use
Alla_P2_S3	GAAGTATGCTGCATTATTTCC		Sequencing primer part 2
Alla_P2_S4	GATCTCTTAAATCTAGGGAGG		Sequencing primer part 2
Alla_P2_S5	CAGAGAAAATCTTTGGAAAGC		Sequencing primer part 2
Alla_P2_S6	CGAAAGTATGGCGTTACTC		Sequencing primer part 2
Alla_P2_S7	GAAGACAAGCATGCAAAT		Sequencing primer part 2
KASP marker DGAT1-2 F469			
DGAT_li_hi	FAM-Tail-GGCAACATGATATTTTGGTTCTTCT		Ins, high OC allele primer
DGAT_li_lo	HEX-Tail-GGCAACATGATATTTTGGTTCTTCA		Del, low OC allele primer
DGAT_li_Com	ACACACATCGGCTGTCCGAC		Common primer
KASP marker allantoinase promoter InDel at -350bp			
Alla_Pro350_li_Del	FAM-Tail-AGCCGCGTCACACACACTCT		Del primer
Alla_Pro350_li_Ins	HEX-Tail-AGCCGCGTCACACACACACA		Ins primer
Alla_Pro350_li_Com	GAGGAAGCCGAAAATCCGAGATTC		Common primer
KASP marker allantoinase C721A (L107M)			
Alla_721_li_A	FAM-Tail-GATTAATTCCTTCAGGCATGCCCATA		A allele primer
Alla_721_li_C	HEX-Tail-GATTAATTCCTTCAGGCATGCCCATC		C allele primer
Alla_721_li_Com	TGCTCTTGTGCCATTGGAGAAGC		Common primer

Primer	Sequence (5’ – 3’)	PCR info	Use
KASP marker allantoinase G3615A (D454N)			
Alla_3615_li_A	FAM-Tail-ACCCGAAACAGAGTTTCAACTTA		A allele primer
Alla_3615_li_G	HEX-Tail-ACCCGAAACAGAGTTTCAACTTG		G allele primer
Alla_3615_li_Com	CAAGTGTTGTAGGGCCATTTG		Common primer
Allantoinase qPCR			
Alla_qPCR_For3	GACTTTCCCATGACAAATTCAACTC	60°C, 55 cycles	Allantoinase qPCR
Alla_qPCR_Rev3	CACGTTGCAGATCTCTGATAGCC		
EF1a_For	TGGGCCTACTGGTCTTACTACTGA		qPCR internal control
EF1a_Rev	ACATACCCACGCTTCAGATCCT		(from Lin et al. 2014)

Table S2 QTL identified for the agronomic traits early vigor, Fusarium resistance, kernel row number, and for the metabolites stigmastan, galacturonic acid and UN_322_870.

	Chr.	Pos. (bp)	π_G	a-Effect
Early vigor	5	689.838	25.2	-0.78
	5	11.812.047	13.3	-1.09
Fusarium resistance	2	15.450.819	18.2	-0.53
	3	29.675.055	5.4	-0.55
Kernel row number	5	55.320.091	10.9	-0.77
Stigmastan	2	227.085.703	20.4	0.09
Galacturonic acid	2	12.177.121	5.1	-0.12
	7	89.268.862	9.4	-0.10
UN_322_870	4	6.234.619	70.3	-0.44

π_G (in %) refers to the explained genotypic variance and a-Effect to the additive effect of the QTL in the panel of doubled haploid lines from all landraces.

Table S3 Effect of the identified F469 polymorphism in *DGAT1-2*. Proportion of explained genotypic variance (π_G in %) and additive effect of the QTL (a-Effect) in the panel of doubled haploid lines from all landraces and in each landrace are shown based on genotyping with the developed KASP marker. In addition, results are shown for marker AX-91354345 from the 50k SNP array. *P* values are derived from the association mixed model analysis with a kinship matrix and the fixed effect for the population.

	DGAT1-2 (F469-)	AX-91354345
Entire landrace panel		
P value	2.01e-10	1.94e-7
Freq.	0.27	0.22
π_G	13.6	10.1
a-Effect	0.22	0.20
Within landraces and elite lines		
Freq. in CG	0.00	0.00
Freq. in GB	0.50	0.29
Freq. in RT	0.43	0.29
Freq. in SF	0.14	0.13
Freq. in SM	0.00	0.00
Freq. in WA	0.51	0.50
Freq. in EF	0.88	0.88
π_G in CG	-	-
π_G in GB	23.1	9.1
π_G in RT	4.1	1.2
π_G in SF	6.9	6.5
π_G in SM	-	-
π_G in WA	33.7	28.8
π_G in EF	21.6	16.4
a-Effect in CG	-	-
a-Effect in GB	0.24	0.16
a-Effect in RT	0.07	0.04
a-Effect in SF	0.17	0.17
a-Effect in SM	-	-
a-Effect in WA	0.28	0.25
a-Effect in EF	0.31	0.27

CG, Campan Galade; GB, Gelber Badischer; RT, Rheintaler; SF, Strenzfelder; SM, Satu Mare; WA, Walliser; EF, elite Flint

For *DGAT1-2* the parameters (allele frequency, π_G and a-Effect) refer to F469, the allele with the phenylalanine insertion causing increased oil and oleic acid concentrations. For marker AX-91354345 [C/T], the same parameters refer to the 'C' allele. This marker is in LD with the F469 polymorphism and the 'C' allele is indicative for the insertion.

Table S4 Polymorphisms in the allantoinase. Sequencing of the allantoinase (gene symbols: Zm00001d026635, GRMZM2G173413, LOC100274212) in a discovery set of elite Flint (EF) and landrace doubled haploid lines. AX-90560856 is a marker identified in the genome-wide scan. The position of non-synonymous polymorphisms is shown relative to the start codon of the reference genome (B73_v4) and in addition the amino acid position and change are shown.

Genotype	Pop	Allantoin content	AX-90560856	-350 TCA/---	A279G I48V	C721A L107M	T1981C S226P	A2037C E244D	G3356A V409I	G3615A D454N
L050	EF	8.271	T	Ins	A	C	T	A	G	G
F169	EF	8.061	C	Del	G	A	C	C	A	A
F110	EF	9.819	C	Del	G	A	C	C	A	A
F160	EF	9.706	C	Del	G	A	C	C	A	A
CAMP.107	CG	8.073	T	Ins	G	C	C	C	A	G
CAMP.105	CG	9.906	C	Del	na	C	C	C	A	A
GELB.A122	GB	8.6246	T	Ins	G	C	C	C	A	na
GELB.122	GB	10.110	C	Del	G	A	C	C	A	A
STGA.104	RT	8.504	T	Ins	A	C	T	A	G	G
STGA.116	RT	10.189	C	Del	G	A	C	C	A	A
STRE.146	SF	7.710	C	Del	G	A	C	C	A	G
STRE.219	SF	9.960	C	Del	G	A	C	C	A	A
SATU.131	SM	7.887	T	Ins	A	C	T	A	G	G
SATU.189	SM	9.999	C	Del	G	C	C	C	A	A
WALL.210	WA	7.590	T	Ins	G	C	C	C	A	A
WALL.165	WA	9.458	C	Del	G	A	C	C	A	G

Conclusions: This table is best interpreted in combination with Supplementary Fig. 7 and the alignment of allantoinase protein sequences from diverse plant species shown in Supplementary Table 5. In general, we found variation in allantoin content in our maize panel and it is likely, that variation is also present in other species. Such variation in allantoin content may be random or linked to an adaptive advantage under certain environmental conditions, resulting in geographical patterns of allantoin content. Thus, different allantoinase alleles may be present in a species and the sequence from a single individual, that by chance may be a high- or a low-allantoin content type, may not be representative for the allelic variation present in that species. Furthermore, the trait is not monogenic, i.e. not only controlled by the allantoinase, such that high and low allantoin content can be found in genotypes carrying the same allantoinase alleles. So, if there is a non-synonymous polymorphism with two different amino acids found at a certain position of the protein in different species, there are two possible interpretations. Either the position is less important and the protein has the same functionality with either of the two amino acids, or the position is essential in conferring allantoinase function and the two amino acids contribute to high or low allantoin content. If an amino acid or even the region around this amino acid is highly conserved across the diverse plant species compared here, this may indicate that the position is important for the protein's functionality and that an amino acid exchange at this position is more likely to alter the allantoinase function or effectivity and thus allantoin content.

As shown in Supplementary Fig. 7, only one elite Flint (EF) line carries the 'T' allele at marker AX-90560856; this line is different from the other EF lines, but at all polymorphic positions. Likewise, for GB only one line and

for RT only two lines carry the presumed low-allantoin ‘T’ allele at marker AX-90560856. For SF only the ‘C’ allele is present; notably, the marker as a proxy for the causal polymorphism being monomorphic does not necessarily mean that there is no variation in the allantoinase in the SF population. For SM, both marker alleles are present with reasonable frequencies, but do not show any difference in allantoin content. The two landrace populations that are likely most informative and may have driven the identification of this QTL are CG and WA, because the marker segregates with almost equal allele frequencies and lines with the different alleles show a clear difference in allantoin content. For these two landraces the non-synonymous mutations L107M and D454N are polymorphic. However, D454N has the ‘G’ for the low allantoin line and ‘A’ for the high allantoin line in the landrace CG, but vice versa for the two WA landrace lines. Moreover, both Asp (D) and Asn (N) as amino acid occur for different plant species (see Supplementary Table 5), indicating that D or N at this position is less critical (unless it causes high and low allantoin levels in different species or different genotypes from a species). L107M matches the pattern of the marker identifying this QTL in WA but not in CG, and is an amino acid that appears to be highly conserved across different plant species. For SM, both lines carry the same allele for L107M, in line with the two marker allelic groups not being different in their allantoin content. However, KASP markers for L107M and D454N were not significantly associated with the trait and explained almost nothing of the genotypic variance, both in contrast to the marker AX-90560856 (see Supplementary Table S7). The InDel in the promoter region at position -350 bp relative to the start codon, by contrast, perfectly followed the allelic pattern of the marker AX-90560856. Notably, several polymorphisms in the promoter region show the same pattern as this InDel in this discovery set of lines (see Supplementary Table S6). We developed a KASP marker for the InDel at -350 as a proxy for the various promoter polymorphisms. This KASP marker was more strongly associated with the trait and explained a higher proportion of genotypic variance. This suggests that one or several polymorphisms in the promoter region or in another regulatory region being in LD with it, contribute to this QTL. Notably, this does not rule out an effect of the non-synonymous polymorphism in the coding region. In fact, different polymorphisms may contribute to the differences in allantoin levels in the different landraces. For example, neither the marker AX-90560856 nor the promoter InDel were polymorphic between the two SF lines, but the non-synonymous mutation D454N, which in this landrace explained 26.0% of the genotypic variation.

Table S5 Alignment of the allantoinase protein sequence from diverse plant species. The positions of the non-synonymous polymorphisms identified in the discovery set of lines are highlighted in yellow.

```
                                                                          I48V
Brachypodium       MAAAAAAAGKGRALPLLAVAAALAAALLYRAP------FSKSLGGEGCSLLPQDHFWIAS       54
Barley             -----MAAGKGRVLPLLAVAAALAAALLYTAP------FSKSLGGEGCSLLPHGHFWIAS       49
Durum              -----MAAGTGRVLPLLAVAAALAAALLYTAP------FSKSLGGEGCSLLPHGHFWIAS       49
Rice               ---MAMAAAKGRVLPLLAVAAALAAALLYRAP------FSKSLGGEGCSLLPHDHFWIAS       51
FoxtailMillet      ----MAASAKGRLVPVLAVVAALAAALLYRAP------FSKSLGGEGCSLLPHDHFWIAS       50
Panicgrass         ----MAASAKGRLVPVLAVAAALAAVLLYRAP------FSKSLGGEGCSLLPHDHFWIAS       50
Maize              ----MAASAKGRLVPVLAVAAALAAVLLYRAP------FSKSLGGEGCSLLPHDHFWIAS       50
Sorghum            ----MVASAKGRLVSVLAVAAALAAVLLYRAP------FSKSLGGEGCSLLPHDHFWVAS       50
Medicago           -----MDQLLWRVLPLLTLLISFSLFFYLQS-------PIIKLPGDECSLLPYSHYWISS       48
Soybean            -----MEQFVWRVLPLLTILMSLVVFFYLQDSYRAHLHPLVKLPRSKCSLLPHRHFWITS       55
Pigeonpea          -----MEQLLWRVLPLLTILVSFIVFFYLQDSYRAQLYPIIKLPGGKCSLLPHRHFWITS       55
Quinoa             -----MEFQKRSFFSLLPVIASILLFFLKFS---------LKTAPSGCSLLHDNHYWISS       46
Tomato             -----MESGKKSTLALLPL---FLSFLFYFDF----S---KKLPSSDCSLLPHNHYWISS       45
Potato             -----MESGKRSTLALLPL---FLSFLFYFDF----S---KKLPSSDCSLLPHNHYWISS       45
Sunflower          -----MESGTWRFLMSFLPLVIYLLFIIYSGY----NSPQPTTTTSRCSLLPHHHYWIAS       51
Cucumber           -----MNLLQWKLLPLLTLLASIF-LFFYL------KDPS----DNECSLLPHKHFWITS       44
Arabidopsis        ---MERTLLQWRLLPLLALIVALF-SFFFA------S-PRSLQGNNKCSLLPHDHYWISS       49
Rapeseed           ---MERSLLQWRLLPLLALIASFL-SVFFA------S--RSYEKETQCSLLPHDHYWISS       48
Apple              MRERKMDALQWRILPVLTLIASFL-LFFYF------QYPSQ-QYPKNCSLLPFQHYWIAS       52
Cotton             -----MDLLHWKLFPLSALLASFL-FFFYI------QDSSK-SSQSGCSLFPHSHYWIAS       47
Jatropha           -----MDNLQWRILPLLALFASFL-FFFYV------QNSSK-LSHGDCSLLPYKHYWITS       47
Populus            -----MDYLQLRVLPLLALLASLI-YFFYF------QDSPTLFERSDCSLFPYNHYWIIS       48
                                .            .                    ***:   *:*: *

                                                                           L107M
Brachypodium       ERVVTLGRVGPAAVEVKGGLINAIAVGDY-RSF-VLRRPLLDYGDAVIMPGLIDVHAHLD       112
Barley             QRVVALGRVGPAAVEVKEGLINAIAVGDY-RSF-VLRRPLVDYGDAVIMPGLIDVHAHLD       107
Durum              QRVVALGRVGPAAVEVKEGLINAIAVGDY-RSF-VLRRPLVDYGDAVIMPGLIDVHAHLD       107
Rice               ERVVTLGRVGPAAVEVKGGLINAIAVGDY-RSF-LLRRPVVDYGDAVIMPGLIDVHAHLD       109
FoxtailMillet      ERVVTLGRVGPAAVEVKGGLINAIAVGDY-RSF-VLRRPLLDYGDAVIMPGLIDVHAHLD       108
Panicgrass         ERVVTLGRVGPAAVEVKGGLINAIAVGDY-RNF-VLRRPLLDYGDAVIMPGLIDVHAHLD       108
Maize              DRVVTLGRVGPAAVEVKGGLINAIAVGDY-RSF-VLRRPLLDYGDAVIMPGLIDVHAHLD       108
Sorghum            DRVVTLGRVGPAAVEVKGGLINAIAVGDY-RSF-VLRRPLLDYGDAVIMPGLIDVHAHLD       108
Medicago           KRILTSQGFISGSVEINEGKIVSIVEGYGKKDNS--VHQVIDYGDAVVMPGLIDVHVHLD       106
Soybean            KRIVTPQGIISGSVEINEGKIISVIKGYSKQGKSK-QEKIIDYGGAVVMPGLIDVHVHLD       114
Pigeonpea          KRIVTPQGIISGSVEINEGRIISVIEGNGKQGKSK-QEEVIDYGDAVVMPGLIDVHVHLD       114
Quinoa             KRIVTQDGVISGAVEIKDGKVFSLVKEEDWHGYIR-KGQIVDFGEAVVMPGLIDVHAHLD       105
Tomato             KRIVTPNGTISGAVEIKEGRIISVVAEENWHVNSR-FTTVVNYRESVVMPGFIDVHAHLD       104
Potato             KRIVTPNGTISGAVEIKEGRIISVVAEENWHVNSS-FTTVVNYGESVVMPGFIDVHAHLD       104
Sunflower          KRIVTPHEIFSGAVEINGGSIISLVKEKDWLERVK-NEPVIDYGEAVVMPGLIDVHAHLD       110
Cucumber           KRIVTPQGVISGAVEINGGKIVSIVKEEEKHGKIM-GNHVVDYADAVVMPGLVDVHVHLD       103
Arabidopsis        KRIVTPNGLISGSVEVKGGIIVSVVKEVDWHKSQRSRVKVIDYGEAVLMPGLIDVHVHLD       109
Rapeseed           KRILTPDGLISGSVEVNGGVIVSVVKEEDWYKEQRSRVKVTDYGEAVIMPGLVDVHAHLD       108
Apple              KRIVTPQGVISGAVEIKDGKIASIVKEEEKHGKIK-SENVIDYGEAVVMPGLIDVHAHLD       111
Cotton             KRIVTPQGIISGAVEIKGGSIVSIVKNKDWSGKFK---QVVDYGNAVVMPGLIDVHAHLD       104
Jatropha           KSIVTPQGIISGAVEVKEGNIVSIVKEEDWQGYSK-RGPILDYGEAVVMPGLIDVHAHLD       106
Populus            KRIVTPHGVISGAVEVKEEKIVSIIKEEDWHGNLK-KGQIIDYGEAVVMPGLIDVHAHLN       107
                   . :::      .:**::   : ::               : ::  :*:***::***.**:

Brachypodium       EPGRAEWEGFSTGTRAAAAGGITTLVDMPLNSFPSTVSEETLKLKVEASRDKLYVDVGFW       172
Barley             EPGRAEWEGFSTGTRAAAAGGITTLVDMPLNSFPSTVSEETLKLKVDAARDKLYVDVGFW       167
Durum              EPGREEWEGFSTGTRAAAAGGITTLVDMPLNSFPSTVSEETLKLKVDAARDKLYVDVGFW       167
Rice               EPGRAEWEGFSTGTRAAAAGGITTLVDMPLNSYPSTVSEETLKLKLDAAKDKLHVDVGFW       169
FoxtailMillet      EPGREEWEGFSTGTKAAAAGGITTLVDMPLNNFPSTVSEETLKLKLEAARDKLYVDVGFW       168
Panicgrass         EPGREEWEGFSTGTRAAAAGGITTLVDMPLNSFPSTVSEETLKLKLEAARDKLFVDVGFW       168
Maize              EPGRAEWEGFSNGTRAAAAGGITTLVDMPLNSFPSTVSEETLKLKLEAARDKLYVDVGFW       168
Sorghum            EPGRAEWEGFSTGTRAAAAGGITTLVDMPLNSFPSTVSEETLKLKLEAAQDKLYVDVGFW       168
Medicago           EPGRTDWEGFVTGTQAAAAGGVTTVVDMPLNNHPTTMSKETLELKLEAAEDKIYVDVGFW       166
Soybean            EPGRTAWEGFDTGTRAAAAGGVTTVVDMPLNNHPTTVSRETLKLKLEAAENKIYVDVGFW       174
Pigeonpea          EPGRTEWEGFDTGTRAAAAGGVTTVVDMPLNNQPTTVSKETLELKLEAAEDKIYVDVGFW       174
Quinoa             DPGREEWEGFPSGTRAAAAGGITTLIDMPLNSEPSTISEETLKLKINAAKNRIFVDVGFW       165
Tomato             DPGRSEWEGFPSGTKAAAAGGVTTLVDMPLNSAPSTVSEETLKLKVQAAEGRVYVDVGFW       164
Potato             DPGRSEWEGFPSGTKAAAAGGVTTLVDMPLNSAPSTVSEETLKLKVQAAEGRVYVDVGFW       164
Sunflower          DPGREEWEGFPSGTRAAAAGGITTLIDMPLNSDPSTVSEETLQLKIKAAKGRIFVNVGFW       170
Cucumber           DPGRSEWEGFPSGTKAAAAGGVTTLVDMPLNNFPSTTSEETLKLKIKAAEGRIYVDVGFW       163
Arabidopsis        DPGRSEWEGFPSGTKAAAAGGITTLVDMPLNSFPSTVSPETLKLKIEAAKNRIHVDVGFW       169
Rapeseed           DPGRSEWEGFPSGTKAAAAGGITTLIDMPLNSDPSTVSPETLKLKIEAAKERILVDVGFW       168
Apple              DPGRAEWEGFPSGTKAAAAGGITTLVDMPLNSDPSTVSRETLELKIKAAESRIYVDVGFW       171
Cotton             DPGRAEWEGFPSGTKAAAAGGVTTLIDMPLNNFPSTVSTETLKLKIEAAEKSIYVDVGFW       164
Jatropha           DPGRTEWEGFPSGTKAAAAGGITTLIDMPLNSFPSTVSVETFRLKLEAAEKNIYVDVGFW       166
```

```
Populus            DPGRTEWEGFPSGTRAAAAGGITTLIDMPLNNFPSTVSAETLKLKIDAAEKNIYVDVGFW       167
                   :***  ****  .**:******:**::*****. *:* * **:.**:.*:.  : *:****

                                                                      S226P
Brachypodium       GGLVPENALNPSKLESLLNAGVLGLKSFMCPSGINDFPMTNSTHIEEGLVTLAKYKRPLL       232
Barley             GGLVPENALNPSKLESLLNAGVLGLKSFMCPSGINDFPMTNSTHIEEGLVTLAKYKRPLL       227
Durum              GGLVPENALNPSKLESLLNAGVLGLKSFMCPSGINDFPMTNSTHIEEGLVTLAKYKRPLL       227
Rice               GGLVPENALNPSALESLLNAGVLGLKSFMCPSGINDFPMTNSTHIEEGLVTLAKYKRPLL       229
FoxtailMillet      GGLVPENAFNPSALESLLNAGVLGLKSFMCPSGINDFPMTNSTHIEEGLVTLAKYQRPLL       228
Panicgrass         GGLVPENAFNPSALESLLNAGVLGLKSFMCPSGINDFPMTNSTHIEEGLVTLAKYQRPLL       228
Maize              GGLVPENAFNPSALEGLLKAGVLGLKSFMCPSGINDFPMTNSTHIEEGLVTLAKYKRSLL       228
Sorghum            GGLVPENAFNPSALEGLLNAGVLGLKSFMCPSGINDFPMTNSTHIEEGLVTLAKYKRPLL       228
Medicago           GGLVPENAQNTSILEGLLDAGVLGLKSFMCPSGIDDFPMTTIEHIKEGLSVLAKYRRPLL       226
Soybean            GGLIPENAHNTSILEGLLSAGVLGLKSFMCPSGISDFPLTTIHHIKEGLSVLAKYRRPLL       234
Pigeonpea          GGLIPENALNTSILEGLLSAGVLGVKSFMCPSGIDDFPMTTIDHIKEGLSVLARYRRPLL       234
Quinoa             GGLVPDNALNATALERFLRAGVLGLKSFMCPSGINDFPMTNITHIKEGLSVLAKFKRPLL       225
Tomato             GGLVPENAENTSSLERLLNAGVLGLKSFMVPSGINDFPMTTASHIKEALPTLARYKRPLL       224
Potato             GGLVPENAENASSLERLLNAGVLGLKSFMVPSGINDFPMTTASHIKEALPTLARYKRPLL       224
Sunflower          GGLVPENAFNATILQNLLNAGALGLKSFMCPSGINDFPMTDASHIKEGLSVLAKYKRPLL       230
Cucumber           GGLVPENAFNASALENLLKAGALGLKSFMCPSGINDFPMTNITHIKEGLSVLAKYKRPLL       223
Arabidopsis        GGLVPDNALNSSALESLLDAGVLGLKSFMCPSGINDFPMTNITHIKEGLSVLAKYKRPLL       229
Rapeseed           GGLVPDNALNSSALSSLLDAGVLGLKSFMCPSGINDFPMTNITHIKEGLSVLAKYKRPLL       228
Apple              GGLVPENAFNASALEDLLDAGVLGLKSFMCPSGINDFPMTNASHIKEGLSVLAKYRRPLL       231
Cotton             GGLVPENAFNATALEALLDAGVHGLKSFMCPSGIDDFPMTDASHIKSGLSVLAKYKRPLL       224
Jatropha           GGLVPENAFNASSLEALLNAGVLGLKSFMCPSGINDFPMTNARHIKEGLSTLAKYRRPLL       226
Populus            GGLVPENAFDANSLEALLSAGALGLKSFMCPSGINDFPMTNSSHIKEGLSILAKYRRPLL       227
                   ***:*:** : . *. :* **. *:**** ****.***:*   **:..*  **:::* **

                                 E244D
Brachypodium       IHAERIPDVESDEGI-DGELDPRSYSTYLKSRPPIWEESAIRDLQRAMKDTKVGGRSEGA       291
Barley             IHAERIPDVASDEEL-DGELDPRSYATYLKSRTPAWEESAVRDLKRAMKDTEVGGRSEGA       286
Durum              IHAERIPDVSSDEEL-DGELDPRSYATYLKSRTPAWEESAVRDLNRAMKDTEVGGRSEGA       286
Rice               IHAERIPDVQNEDGI-DGELDPKAYTTYLKSRPPAWEEAAIKDLQRAMKDTEIGGRSEGA       288
FoxtailMillet      VHAERIPDVEDEDGL-DGELDPRSYATYLKSRPPAWEESAIRDLQRAMKDTEAGGRSEGA       287
Panicgrass         IHAERIPDDEDDDGL-DGELDPRSYATYLKSRPPAWEESAIRDLQRAMKDTEAGGRSEGA       287
Maize              IHAERIPEAEDHDLQ-EDELDPRSYMTYLKSRPPSWEEAAIRDLQRAMKDTELGGRSEGA       287
Sorghum            IHAERIPEAEDDDMR-EDELDPRSYMTYLKSRPPSWEESAIRDLHRAMKDTESGGRSEGA       287
Medicago           VHSEIQQDSESSSELEG-NGDPHSYKTYLDTRPPSWEEAAIKELVDATKDTRIGGSLEGA       285
Soybean            VHSEIQQHSKKHLELND-KGGPRAYLTYLHTRPPSWEEAAIKELVGVTKDTRKGGPLEGA       293
Pigeonpea          VHSEIQQDSENRLELND-NRDPHDYLTYLNTRPPSWEQAAITELVGVTKDTRIGGPLEGA       293
Quinoa             VHAEVQQDFADK-SVKDDKKDARLYSTYLETRPPSMEEAAIRNLLTVSENTRAGGPAEGA       284
Tomato             VHAEVLVDLDEKVELEDGVENARSYSTYLKTRPASMEEAAINQLITLSKDARAGGSAEGA       284
Potato             VHAEVLLDLDGKVELEDGVENARSYSTYLKTRPASMEEAAINQLITLSKDTRAGGSAEGA       284
Sunflower          VHAEKQQEVE----LKDGDDDPRSYSTYLKTRPSSMEETAIRELLTVAKDTRTGGPAEGA       286
Cucumber           VHSEIEQSSPSPVQLEGSQDDPRTYSTYLATRPPSWEEAAVRELLKVTSNTRPGGPAEGA       283
Arabidopsis        VHAEIERDLEIE---DGSENDPRSYLTYLKTRPTSWEEGAIRNLLSVTENTRIGGSAEGA       286
Rapeseed           VHAEVEMDSEID---DGTDNDPRSYLTYLKTRPSSWEEGAIRNLLSVTENTRIGGSAEGA       285
Apple              VHAEKEQELESNLGVEDGSNNPRSYSTYLKTRPPSWEEAAIKDLLTLTKDTRIGGPAEGA       291
Cotton             VHSEIQSVVESHLETEDGGGDPRSYSTYLKTRPPSWEEAAIRELLTVTKDTRSGGPAEGA       284
Jatropha           VHAEIQQDSENY--TEDAENDPRHYSTYLKTRPPSWEEAAIRDLVTLSKDTRIGGPAEGA       284
Populus            VHAEIPQDSESH--LEDGENDPRSYSTYLKTRPPSWEEAAIRELLTVSKDTRIGGPAEGA       285
                   :*:*                . : * *** :*    *: *: :*    .::. **  ***

Brachypodium       HIHIVHLSDAETSLQLLKDAKHNGARVSIETCPHYLAFSAEEVPDGDTRFKCSPPIRHAA       351
Barley             HLHIVHLSDSKTSLRLLKDAKHSGAKVTIETCPHYLAFSAEEVPDGDTRFKCSPPIRDAA       346
Durum              HLHIVHLSDSKTTLDLLKDAKHSGAKVTIETCPHYLAFSAEEVPDGDTRFKCSPPIRDAA       346
Rice               HIHIVHLSDAKTSLGLLKDAKQNGARVSVETCPHYLAFSAEEVPDGDTRFKCAPPIRDST       348
FoxtailMillet      HIHIVHLSDAKTSLELMKDAKRTGASVTVETCPHYLAFSADEVPDGDTRFKCAPPIRDGM       347
Panicgrass         HIHIVHLSDAKASLELMKDAKRTGASVTVETCPHYLAFSADEVPDGDTRFKCAPPIRDGM       347
Maize              HIHIVHLSDAKTSLELMKDAKRSGASLSIETCPHYLAFSSEEVPDGDTRFKCSPPIRDDT       347
Sorghum            HIHIVHLSDAKTSLELMKDAKRSGASLSIETCPHYLAFSSEEVPDGDTRFKCSPPIRDDT       347
Medicago           HVHIVHLSDSSASLDLIKEAKRRGDSISIETCPHYLTFSSEEIPDKDTRYKCSPPIRDAS       345
Soybean            HVHVVHLSDSSASLDLIKEAKRRGDSISVETCPHYLAFSSEEIPNGDTRFKCSPPIRDAY       353
Pigeonpea          HVHIVHLSDSSASLDLIKEAKNRGDSLSVETCPHYLAFSSEEIPNGDTRFKCSPPIRDAF       353
Quinoa             HLHIVHLSDAQSSLNLLKNAKARGDSISIETCPHYLSFSADEIPDGDTRFKCAPPIRDAE       344
Tomato             HLHIVHLSDARTSLNLIKEAKQRGDSITVETCPHYLAFAAEDIPDGDTRFKCAPPIRDAA       344
Potato             HLHIVHLSDARTSLNLIKEAKQRGDSITVETCPHYLAFAAEDIPDGDTRFKCAPPIRDAA       344
Sunflower          HVHIVHLSDSRSSLELIKEAKSNGDSVTVETCPHYLAFSAEEIPDGDTRFKCAPPIREAA       346
Cucumber           HIHVAHLSDSGSTLELIKEAKRSGDSVSVETCTHYLAFSEEDIKDGDTRFKCAPPIRDKA       343
Arabidopsis        HLHIVHLSDASSSLDLIKEAKGKGDSVTVETCPHYLAFSAEEIPEGDTRFKCSPPIRDAA       346
Rapeseed           HVHIVHLSDASSSLEMIKEAKGKGDSVTVETCPHYLAFSAEEIPEGDTRFKCSPPIRHAA       345
Apple              HLHIVHLSDSRSSLDLIKEAKSGGDSLTVETCPHYLAFSAEEIPDGDTRFKCSPPIRDAA       351
Cotton             HLHVVHLSDASSSLDLIKEAKRRGDSITVETCPHYLAFSAEEIPDGDTRFKCAPPIRDAA       344
Jatropha           HLHIVHLSDSGSSLQLIKLAKRSGDSVTVETCPHYLAFSAEEIKDGDTRFKCAPPIRDSA       344
Populus            HLHVVHLSDAGSSLQLLKEAKSNGDSVTVETCPHYLAFSAEEIKDGDTRFKCAPPIRDAA       345
                   *:*:.****: ::* ::* **  *  :::*** ***:*: ::: : ***:**:****.

Brachypodium       NKDNLWEALLDGHIDMLSSDHSPSTPDLKLMEDGNFLKAWGGISSLQFVLPVTWSYGKKH       411
```

```
Barley           NKENLWEALLDGHIDMLSSDHSPSTPDLKLMEEGNFMKAWGGISSLQFVLPVTWSHGKKY      406
Durum            NKENLWEALLDGHIDMLSSDHSPSTPDLKLMEEGNFMKAWGGISSLQFVLPVTWSHGKKY      406
Rice             NRDNLWEALLDGHIDMLSSDHSPSAPDLKLMEEGNFLRAWGGISSLQFVLPVTWSHGKKY      408
FoxtailMillet    NRENLWKALLDGHIDMLSSDHSPSAPDLKLMEEGNFLKAWGGISSLQFVLPVTWSYGKKY      407
Panicgrass       NRENLWKALLDGHIDMLSSDHSPSAPDLKLMEEGNFLKAWGGISSLQFVLPVTWSYGKKY      407
Maize            NRENLWKALLDGHIDMLSSDHSPSTPDLKLMEEGDFLRAWGGISSLQFVLPVTWSYGRKY      407
Sorghum          NRENLWKALLDGHIDMLSSDHSPSTPDLKLMEEGNFLRAWGGISSLQFVLPVTWSYGRKY      407
Medicago         NREKLWEALLDGHIDLLSSDHSPTVPKLKLLKEGDFLKAWGGISSLQFDLPVTWSYGKKH      405
Soybean          NREKLWEAVLEGHIDLLSSDHSPTVPQLKLLEEGDFLKAWGGVTSLQFDLPVTWSYGKKH      413
Pigeonpea        NKEKLWEAVLEGDIDLLSSDHSPTVPELKLLEEGDFLRAWGGISSLQFDLPVTWSYGKKY      413
Quinoa           NKEKLWEALRGGDIDMLSSDHSPALPDLKLLQQGDFLKAWGGISSLQYVLPATWTSGRKY      404
Tomato           NKEKLWDALLDGDIDMLSSDHSPSVPEMKLLDEGDFLKAWGGISSLQFVLPVTWTHGRKY      404
Potato           NKEKLWDALLDGDIDMLSSDHSPSVPEMKLLDEGDFLKAWGGISSLQFVLPATWTHGRKY      404
Sunflower        NRQKLWEALMDGDIDMLSSDHSPSEPELKLFAEGDFLRAWGGISSLQFVLPVTWSYGVKY      406
Cucumber         NKEKLWDALMEGHIDMLSSDHSPTVPHLKLPDSGDFLKAWGGVSSLQFDLSATWSHAKKR      403
Arabidopsis      NREKLWEALMEGDIDMLSSDHSPTKPELKLMSDGNFLKAWGGISSLQFVLPITWSYGKKY      406
Rapeseed         NREKLWEAMMEGDIDMLSSDHSPTKPELKLLSDGNFLKAWGGISSLQFVLPVTWSYGRKY      405
Apple            NKEKLWEALLEGHVDMLSSDHSPTMPELKLLDGGDFLKAWGGISSLQFVLPVTWSYGQKY      411
Cotton           NKEKLWNALMEGDIDMLSSDHSPTVPELKLLNDGNFLKAWGGISSIQFVLPVTWSYGQKY      404
Jatropha         NKEALWKALLGGDIDMLSSDHSPTVPELKLFNEGNFLRAWGGISSLQFVLPVTWSYGRKY      404
Populus          NKERLWTALLEGDIDMLSSDHSPSVPDLKLFDEGNFLKAWGGISSLQFVLPVTWSHGRQY      405
                 *:: ** *:  *.:*:*******: *.:**   *:*::****::*:*: *  **: . :

                V409I                                        D454N
Brachypodium     GITLNQLASWWSEKPAMLAGQKNKGSILPGYHADIVVWKPEAEFQLDNSHAVYHKHRNIS      471
Barley           GITLNQLASWWSEKPAELAGQKNKGSILPGYHADIVVWNPEAQFHLDDSHAVYHKHRNIS      466
Durum            GITLNQLASWWSEKPAELAGQKNKGSILPGYHADIVVWKPEAQFHLDDTHAVYHKHRNIS      466
Rice             GISLNQLASWWSERPAMLAGLKKKGAVLPGYRADIVVWKPEAQFHLDDSHPVYHKHRNIS      468
FoxtailMillet    GITLNQLAAWWSENPAKLAGQKNKGAILPGYHADIVVWKPEAQFELDDSHSVYHKHRNIS      467
Panicgrass       GITLNQLAAWWSENPAKLAGQKNKGAILPGYHADIVVWKPEAQFELDDSHSVYHKHRNIS      467
Maize            GVTLNQLASWWSERPAKLAGQKNKGAILPGYHADIVVWKPETEFQLDNNHAIYHKHQNIS      467
Sorghum          GITLNQLSSWWSENPAKLAGQKNKGAILPGYHADIVVWKPETEFQLDDSHVIYHKHQNIS      467
Medicago         GLTLEKLSLLWSQKPAKLAGLESKGAIAVGNHADIVVWQPEVEFDLNDDYPVFIKHPSLS      465
Soybean          GLTLEQLSLLWSKKPATFAGLESKGAIAVGNHADIVVWKPEVEFDLNEDYPVFLKHPSLS      473
Pigeonpea        GLTLEQLSILWSKKPAAFAGLESKGAIAVGNHADIVVWHPELEFDLNDDHPVFLKHPSLS      473
Quinoa           GVTFEQIASWWSEKPAKLAGLPLKGSIAVGNYADIVAWEPETDFDLDQNHPSYFKHPSIS      464
Tomato           GITYEQLASWWSEKPAKLAGLTTKGAIAVGNQADIVVWEPDMEFDLDNDYPVHIKHPSIS      464
Potato           GITFEQLASWWSENPAKLAGLTTKGAIAVGNQADIVVWEPDMEFDLDNDYPVHIKHPSIS      464
Sunflower        GITLEKLVSWWSEKPAKLASQDLKGAIEIGKHADIVVWEPEKEFDLDENHTVHLKHPSIS      466
Cucumber         GVTMEQIALWWSERPAKLAGLELKGAIAIGKHADIVAWAPDEEYDVN-DIPVYLKHPSIS      462
Arabidopsis      GVTLEQVTSWWSDRPSKLAGLHSKGAVTVGKHADLVVWEPEAEFDVDEDHPIHFKHPSIS      466
Rapeseed         GVTLEQVASWWSDRPSKLAGLHSKGAIAVGKHADIVVWEPEVEFDLDEDHPIHFKHPSIS      465
Apple            GVTLQQLALWWSERPARLAGQKLKGAIAVGNHADIVVWDPDVEFDLDDEFPMHVKHPGIS      471
Cotton           GITLEQLVSWWSERPAKLAGQHSKGAIAIGNHADVVVWEPEVEFDLNEDHPMFVKNPSIS      464
Jatropha         GVSLEQLALWWSERPAKLAGQDSKGAIVVGNDADIVIWEPSTVFDLNNDLPIYFKHPSIS      464
Populus          GVTLEQLALWWSERPAKLAGQDLKGAIAVGNHADITVWEPNVEFELNNDLPVYLKHPSIS      465
                 *:: :::   **..*: :*.   **::  *  **:. * *.  :.::     . *: .:*

Brachypodium     AYLGKELSGKVLSTFVRGNLVFAEGKHAGAACGATILAK---      510
Barley           AYLGNELSGKVLSTFVRGNLVFAEGKHAGAACGATILAK---      505
Durum            AYLGKELSGKVLSTFVRGNLVFAEGKHAGAACGATILAK---      505
Rice             AYLGKQLSGKILSTFVGGNLVFAEDKHAKAACGAPILAK---      507
FoxtailMillet    AYLGKELSGKVLSTFVRGNLVFAEDKHAKAACGVQILAK---      506
Panicgrass       AYLGKELSGKVLSTFVRGNLVFAEDKHAKAACGATILAK---      506
Maize            AYLGKQLSGKVLSTFVRGNLVFAEDKHANAACGVPILAK---      506
Sorghum          AYLGKQLSGKVLSTFVRGNLVFAEDKHANVACGVPILAK---      506
Medicago         AYMGSRLAGKVLDTFVRGNLVFKDGKHAPAACGVPILAK---      504
Soybean          AYMGRRLSGKVLETFVRGNLVFKKGKHAPSACGVPILAK---      512
Pigeonpea        AYMGKRFSGKVLDTFVRGNLVFKDGKHAPAACGVPILAK---      512
Quinoa           AYVGRKLSGKVLATFVRGNLVYKEGKHAPAACGNRLLAK---      503
Tomato           AYMGSRLSGKVLATFVGGNLVYKEGNHASQACALPILHK---      503
Potato           AYMGSRLSGKVLATFVRGNLVYKEGNHASQACALPILHR---      503
Sunflower        AYMGSRLAGKVVATFVNGNLVFEGGKHAPDACGTTILA----      504
Cucumber         AYMGMKLSGKVLATFVRGQLVYEE-KHAPAACGTPILARVTD      503
Arabidopsis      AYLGRRLSGKVVSTFVRGNLVFGEGKHASDACGSLQLATT--      506
Rapeseed         AYLGRKLSGKVVSTFVRGNLVFGEGKHASDACGSLLLAT---      504
Apple            AYLGTKLSGRVAATFVRGNLVFEEGKHAPAVCGVPILAS---      510
Cotton           AYIGKRLSGKVLATFVRGNLVYKEGNHAFAACGSTILAT---      503
Jatropha         AYMGTKLSGKVLATFVRGNLVYKEGKHASAACGSLILAK---      503
Populus          AYMGSKLSGKVMSTFVRGNLVYKEGKHAPAACGAPILAT---      504
                 **:* .::*::  *** *:**:   :**  .*.   *
```

Protein sequences used for the alignment: Brachypodium (KQJ85338.1), Barley (BAJ96591.1), Durum (VAH39005.1), Rice (sp|B9FDB8.1|ALN_ORYSJ), Foxtail millet (RCV14868.1), Hall’s panicgrass (PAN40784.1), Maize (NP_001142056.1), Sorghum (EES11665.1), Medicago (AES63773.1), Soybean

(XP_025980880.1), Pigeonpea (KYP62933.1), Quinoa (XP_021740572.1), Tomato (XP_004232860.1), Potato (XP_006347056.1), Sunflower (XP_022036017.1), Cucumber (XP_004146596.1), Arabidopsis (NP_567276.1), Rapeseed (XP_013671907.1), Apple (RXH99206.1), Cotton (KHG26051.1), Jatropha (XP_012091585.1), Populus (XP_002305806.3).

Table S6 Polymorphisms in the allantoinase promoter. Alignment of the promoter sequences of the lines from the discovery set (see Supplementary Table S4). The sequence corresponds to B73_RefGen_v4 Chr10:149244482..149145854, with the last three nucleotides corresponding to the ATG of the allantoinase. The coding of the genotypes is composed of the genotype name, followed by ‘Lo’ or ‘Hi’ for low or high allantoin content, respectively, and ‘T’ or ‘C’ for the allele at marker AX90560856. The TCA Indel at -350 (which may as well be an ATC Indel) that is tagged by the developed KASP marker is indicated. Notably, several polymorphisms upstream of this InDel show the same pattern.

```
Maize_B73_RefGen_v4    ATCGGGGATCATTTCCCCGACAGTAG-CCCCGAGCCCCTG--------AG 50
L050_Lo_T              ATCGGGGATCATTTCCCCGACAGTAG-CCCCGAGCCCCTG--------AG 50
CAMP.107_Lo_T          ATCGGGGATCATTTCCTCAACGGTAGCCCCCGAGCCCCAGGGTTAAAGAG 50
GELB.A122_Lo_T         -------------------------------------------------- 50
STGA.104_Lo_T          ATCGGGGATCATTTCCCCGACAGTAG-CCCCGAGCCCCTG--------AG 50
SATU.131_Lo_T          ATCGGGGATCATTTCCCCGACAGTAG-CCCCGAGCCCCTG--------AG 50
WALL.210_Lo_T          ACCGGGGATCATTTCTCCGACAGTAGCCCCCGAGCCCCTG--------AG 50
F169_Lo_C              ACCGGGGATCATTTCTCCGACAGTAGCCCCCGAGCCCCTG--------AG 50
STRE.146_Lo_C          ACCGGGGATCATTTCTCCGACAGTAGCCCCCGAGCCCCTG--------AG 50
F110_Hi_C              ACCGGGGATCATTTCTCCGACAGTAGCCCCCGAGCCCCTG--------AG 50
F160_Hi_C              ACCGGGGATCATTTCTCCGACAGTAGCCCCCGAGCCCCTG--------AG 50
CAMP.105_Hi_C          ACCGGGGATCATTTCTCCGACAGTAGCCCCCGAGCCCCTG--------AG 50
GELB.122_Hi_C          ACCGGGGATCATTTCTCCGACAGTAGCCCCCGAGCCCCTG--------AG 50
STGA.116_Hi_C          ACCGGGGATCATTTCTCCGACAGTAGCCCCCGAGCCCCTG--------AG 50
STRE.219_Hi_C          ACCGGGGATCATTTCTCCGACAGTAGCCCCCGAGCCCCTG--------AG 50
SATU.189_Hi_C          ACCGGGGATCATTTCTCCGACAGTAGCCCCCGAGCCCCTG--------AG 50
WALL.165_Hi_C          ACCGGGGATCATTTCTCCGACAGTAGCCCCCGAGCCCCTG--------AG 50

Maize_B73_RefGen_v4    GGTTTGATTGTGGCTGCTTAAGTTGTAATCATCCATCCCTTGCCAATTGA 100
L050_Lo_T              GGTTTGATTGTGGCTGCTTAAGTTGTAATCATCCATCCCTTGCCAATTGA 100
CAMP.107_Lo_T          GGTTTGATTGGGACTGC-TAAGCTGTAATCGTCCATCCCTTGTCAATTGA 100
GELB.A122_Lo_T         -------------------------------------------------- 100
STGA.104_Lo_T          GGTTTGATTGTGGCTGCTTAAGTTGTAATCATCCATCCCTTGCCAATTGA 100
SATU.131_Lo_T          GGTTTGATTGTGGCTGCTTAAGTTGTAATCATCCATCCCTTGCCAATTGA 100
WALL.210_Lo_T          GG--TGATTGGGACTGTTTAAACTGTAATCATCCATCCCTTGCCAATTGA 100
F169_Lo_C              GG--TGATTGGGACTGTTTAAACTGTAATCATCCATCCCTTGCCAATTGA 100
STRE.146_Lo_C          GG--TGATTGGGACTGTTTAAACTGTAATCATCCATCCCTTGCCAATTGA 100
F110_Hi_C              GG--TGATTGGGACTGTTTAAACTGTAATCATCCATCCCTTGCCAATTGA 100
F160_Hi_C              GG--TGATTGGGACTGTTTAAACTGTAATCATCCATCCCTTGCCAATTGA 100
CAMP.105_Hi_C          GG--TGATTGGGACTGTTTAAACTGTAATCATCCATCCCTTGCCAATTGA 100
GELB.122_Hi_C          GG--TGATTGGGACTGTTTAAACTGTAATCATCCATCCCTTGCCAATTGA 100
STGA.116_Hi_C          GG--TGATTGGGACTGTTTAAACTGTAATCATCCATCCCTTGCCAATTGA 100
STRE.219_Hi_C          GG--TGATTGGGACTGTTTAAACTGTAATCATCCATCCCTTGCCAATTGA 100
SATU.189_Hi_C          GG--TGATTGGGACTGTTTAAACTGTAATCATCCATCCCTTGCCAATTGA 100
WALL.165_Hi_C          GG--TGATTGGGACTGTTTAAACTGTAATCATCCATCCCTTGCCAATTGA 100

Maize_B73_RefGen_v4    TTATGAGTTGTTTTTACCTAAAGTGGAAGTGAACTGTGCAAAAGCTTGTA 150
L050_Lo_T              TTATGAGTTGTTTTTACCTAAAGTGGAAGTGAACTGTGCAAAAGCTTGTA 150
CAMP.107_Lo_T          TTCCGACTTGTTTTTATCTAATGTGGAAGTGAACTGTGCAAAAGCTTGTA 150
GELB.A122_Lo_T         -------------------------------------------------- 150
STGA.104_Lo_T          TTATGAGTTGTTTTTACCTAAAGTGGAAGTGAACTGTGCAAAAGCTTGTA 150
SATU.131_Lo_T          TTATGAGTTGTTTTTACCTAAAGTGGAAGTGAACTGTGCAAAAGCTTGTA 150
WALL.210_Lo_T          TTCTGACTTGTTTTTACCTAAAGTGGAAGTGCACTGTGCAAAAGCTTGTA 150
F169_Lo_C              TTCTGACTTGTTTTTACCTAAAGTGGAAGTGCACTGTGCAAAAGCTTGTA 150
STRE.146_Lo_C          TTCTGACTTGTTTTTACCTAAAGTGGAAGTGCACTGTGCAAAAGCTTGTA 150
F110_Hi_C              TTCTGACTTGTTTTTACCTAAAGTGGAAGTGCACTGTGCAAAAGCTTGTA 150
F160_Hi_C              TTCTGACTTGTTTTTACCTAAAGTGGAAGTGCACTGTGCAAAAGCTTGTA 150
CAMP.105_Hi_C          TTCTGACTTGTTTTTACCTAAAGTGGAAGTGCACTGTGCAAAAGCTTGTA 150
GELB.122_Hi_C          TTCTGACTTGTTTTTACCTAAAGTGGAAGTGCACTGTGCAAAAGCTTGTA 150
```

```
STGA.116_Hi_C          TTCTGACTTGTTTTTACCTAAAGTGGAAGTGCACTGTGCAAAAGCTTGTA 150
STRE.219_Hi_C          TTCTGACTTGTTTTTACCTAAAGTGGAAGTGCACTGTGCAAAAGCTTGTA 150
SATU.189_Hi_C          TTCTGACTTGTTTTTACCTAAAGTGGAAGTGCACTGTGCAAAAGCTTGTA 150
WALL.165_Hi_C          TTCTGACTTGTTTTTACCTAAAGTGGAAGTGCACTGTGCAAAAGCTTGTA 150
Maize_B73_RefGen_v4    ATTCTGTGGATTGAGCTTGTTCCAATTTTGATTTGCATGGATACCAGTTC 200
L050_Lo_T              ATTCTGTGGATTGAGCTTGTTCCAATTTTGATTTGCATGGATACCAGTTC 200
CAMP.107_Lo_T          ATTACGTGGATTGAGCTTGTTCTAATTTTGATTTGCATGGATACCAGTTC 200
GELB.A122_Lo_T         -------------------------------------------------- 200
STGA.104_Lo_T          ATTCTGTGGATTGAGCTTGTTCCAATTTTGATTTGCATGGATACCAGTTC 200
SATU.131_Lo_T          ATTCTGTGGATTGAGCTTGTTCCAATTTTGATTTGCATGGATACCAGTTC 200
WALL.210_Lo_T          ATTCTGTGGATTGAGCTTGTTCCAATTTTGATTTGCATGGATACCATTTC 200
F169_Lo_C              ATTCTGTGGATTGAGCTTGTTCCAATTTTGATTTGCATGGATACCAGTTC 200
STRE.146_Lo_C          ATTCTGTGGATTGAGCTTGTTCCAATTTTGATTTGCATGGATACCAGTTC 200
F110_Hi_C              ATTCTGTGGATTGAGCTTGTTCCAATTTTGATTTGCATGGATACCAGTTC 200
F160_Hi_C              ATTCTGTGGATTGAGCTTGTTCCAATTTTGATTTGCATGGATACCAGTTC 200
CAMP.105_Hi_C          ATTCTGTGGATTGAGCTTGTTCCAATTTTGATTTGCATGGATACCAGTTC 200
GELB.122_Hi_C          ATTCTGTGGATTGAGCTTGTTCCAATTTTGATTTGCATGGATACCAGTTC 200
STGA.116_Hi_C          ATTCTGTGGATTGAGCTTGTTCCAATTTTGATTTGCATGGATACCAGTTC 200
STRE.219_Hi_C          ATTCTGTGGATTGAGCTTGTTCCAATTTTGATTTGCATGGATACCAGTTC 200
SATU.189_Hi_C          ATTCTGTGGATTGAGCTTGTTCCAATTTTGATTTGCATGGATACCAGTTC 200
WALL.165_Hi_C          ATTCTGTGGATTGAGCTTGTTCCAATTTTGATTTGCATGGATACCAGTTC 200


Maize_B73_RefGen_v4    CAAAAGTCAATATGTTAAGTGCTGGTACGTTTCCCTGAAAGTATGTTACC 250
L050_Lo_T              CAAAAGTCAATATGTTAAGTGCTGGTACGTTTCCCTGAAAGTATGTTACC 250
CAMP.107_Lo_T          CAAAAGTCAATATGTTAAGTGCCGGTGCGTTTCCCTTAAAGTATGTCA-C 250
GELB.A122_Lo_T         -------------------------------------------------- 250
STGA.104_Lo_T          CAAAAGTCAATATGTTAAGTGCTGGTACGTTTCCCTGAAAGTATGTTACC 250
SATU.131_Lo_T          CAAAAGTCAATATGTTAAGTGCTGGTACGTTTCCCTGAAAGTATGTTACC 250
WALL.210_Lo_T          CAGAAGTCAATATGTTAAGTGCTGGTGCGTTTCCGTGAAAGTATGCTACC 250
F169_Lo_C              CAGAAGTCAATATGTTAAGTGCTGGTGCGTTTCCGTGAAAGTATGCTACC 250
STRE.146_Lo_C          CAGAAGTCAATATGTTAAGTGCTGGTGCGTTTCCGTGAAAGTATGCTACC 250
F110_Hi_C              CAGAAGTCAATATGTTAAGTGCTGGTGCGTTTCCGTGAAAGTATGCTACC 250
F160_Hi_C              CAGAAGTCAATATGTTAAGTGCTGGTGCGTTTCCGTGAAAGTATGCTACC 250
CAMP.105_Hi_C          CAGAAGTCAATATGTTAAGTGCTGGTGCGTTTCCGTGAAAGTATGCTACC 250
GELB.122_Hi_C          CAGAAGTCAATATGTTAAGTGCTGGTGCGTTTCCGTGAAAGTATGCTACC 250
STGA.116_Hi_C          CAGAAGTCAATATGTTAAGTGCTGGTGCGTTTCCGTGAAAGTATGCTACC 250
STRE.219_Hi_C          CAGAAGTCAATATGTTAAGTGCTGGTGCGTTTCCGTGAAAGTATGCTACC 250
SATU.189_Hi_C          CAGAAGTCAATATGTTAAGTGCTGGTGCGTTTCCGTGAAAGTATGCTACC 250
WALL.165_Hi_C          CAGAAGTCAATATGTTAAGTGCTGGTGCGTTTCCGTGAAAGTATGCTACC 250


Maize_B73_RefGen_v4    ACAAAATAGTTGCAACGGTGTGGTCGGTGAATGGTACTACATTACCGCAA 300
L050_Lo_T              ACAAAATAGTTGCAACGGTGTGGTCGGTGAATGGTACTACATTACCGCAA 300
CAMP.107_Lo_T          ACAAAATAGTTGCAACGGTGTGGTCGGTGAATGGTACTGCATTACCGCAA 300
GELB.A122_Lo_T         -------------------------------------------------- 300
STGA.104_Lo_T          ACAAAATAGTTGCAACGGTGTGGTCGGTGAATGGTACTACATTACCGCAA 300
SATU.131_Lo_T          ACAAAATAGTTGCAACGGTGTGGTCGGTGAATGGTACTACATTACCGCAA 300
WALL.210_Lo_T          ACAAAATAGTTGCAACGATGTGCTCGGTGAATGGTACTGCATTACCGCAA 300
F169_Lo_C              ACAAAATAGTTGCAACGATGTGGTCGGTGAATGGTACTGCATTACCGCAA 300
STRE.146_Lo_C          ACAAAATAGTTGCAACGATGTGGTCGGTGAATGGTACTGCATTACCGCAA 300
F110_Hi_C              ACAAAATAGTTGCAACGATGTGCTCGGTGAATGGTACTGCATTACCGCAA 300
F160_Hi_C              ACAAAATAGTTGCAACGATGTGCTCGGTGAATGGTACTGCATTACCGCAA 300
CAMP.105_Hi_C          ACAAAATAGTTGCAACGATGTGGTCGGTGAATGGTACTGCATTACCGCAA 300
GELB.122_Hi_C          ACAAAATAGTTGCAACGATGTGCTCGGTGAATGGTACTGCATTACCGCAA 300
STGA.116_Hi_C          ACAAAATAGTTGCAACGATGTGCTCGGTGAATGGTACTGCATTACCGCAA 300
STRE.219_Hi_C          ACAAAATAGTTGCAACGATGTGCTCGGTGAATGGTACTGCATTACCGCAA 300
SATU.189_Hi_C          ACAAAATAGTTGCAACGATGTGGTCGGTGAATGGTACTGCATTACCGCAA 300
WALL.165_Hi_C          ACAAAATAGTTGCAACGATGTGGTCGGTGAATGGTACTGCATTACCGCAA 300


Maize_B73_RefGen_v4    AAAATGCTTTGTATTCGTTCGATCTGTTTGGTTGGTCTATGTTTGTGAAA 350
L050_Lo_T              AAAATGCTTTGTATTCGTTCGATCTGTTTGGTTGGTCTATGTTTGTGAAA 350
CAMP.107_Lo_T          AAAATGCTTTGTACTCGTCCGATCTGTTTGGTTGGGCTGTGGCTGTGAAA 350
GELB.A122_Lo_T         -------------------------------------------------- 350
STGA.104_Lo_T          AAAATGCTTTGTATTCGTTCGATCTGTTTGGTTGGTCTATGTTTGTGAAA 350
SATU.131_Lo_T          AAAATGCTTTGTATTCGTTCGATCTGTTTGGTTGGTCTATGTTTGTGAAA 350
WALL.210_Lo_T          AAAATGCTTTGTATTCGTTCGATCTGTTTGGTTGGTCTATGGCTGTGAAA 350
```

```
F169_Lo_C             AAAATGCTTTGTATTCGTTCGATCTGTTTGGTTGGTCTATGGCTGTGAAA 350
STRE.146_Lo_C         AAAATGCTTTGTATTCGTTCGATCTGTTTGGTTGGTCTATGGCTGTGAAA 350
F110_Hi_C             AAAATGCTTTGTATTCGTTCGATCTGTTTGGTTGGTCTATGGCTGTGAAA 350
F160_Hi_C             AAAATGCTTTGTATTCGTTCGATCTGTTTGGTTGGTCTATGGCTGTGAAA 350
CAMP.105_Hi_C         AAAATGCTTTGTATTCGTTCGATCTGTTTGGTTGGTCTATGGCTGTGAAA 350
GELB.122_Hi_C         AAAATGCTTTGTATTCGTTCGATCTGTTTGGTTGGTCTATGGCTGTGAAA 350
STGA.116_Hi_C         AAAATGCTTTGTATTCGTTCGATCTGTTTGGTTGGTCTATGGCTGTGAAA 350
STRE.219_Hi_C         AAAATGCTTTGTATTCGTTCGATCTGTTTGGTTGGTCTATGGCTGTGAAA 350
SATU.189_Hi_C         AAAATGCTTTGTATTCGTTCGATCTGTTTGGTTGGTCTATGGCTGTGAAA 350
WALL.165_Hi_C         AAAATGCTTTGTATTCGTTCGATCTGTTTGGTTGGTCTATGGCTGTGAAA 350

Maize_B73_RefGen_v4   AAAACTGTTGTTGG------CTGTGAGATATAAAAAAA-CTGTCATGAGC 400
L050_Lo_T             AAAACTGTTGTTGG------CTGTGAGATATAAAAAAA-CTGTCATGAGC 400
CAMP.107_Lo_T         AAAAATGTTGTTGGCTGTGACTGTGAGATGTAAAAAAA-TTGTCATGAGT 400
GELB.A122_Lo_T        -------------------------------------------------- 400
STGA.104_Lo_T         AAAACTGTTGTTGG------CTGTGAGATATAAAAAAA-CTGTCATGAGC 400
SATU.131_Lo_T         AAAACTGTTGTTGG------CTGTGAGATATAAAAAAA-CTGTCATGAGC 400
WALL.210_Lo_T         AAAATTGTTGTTGG------CTGTGCGATATAAAAAAAGGTGTCA----- 400
F169_Lo_C             AAAATTGTTGTTGG------CTGTGCGATATAAAAAAAGGTGTCA----- 400
STRE.146_Lo_C         AAAATTGTTGTTGG------CTGTGCGATATAAAAAAAGGTGTCA----- 400
F110_Hi_C             AAAATTGTTGTTGG------CTGTGCGATATAAAAAAAGGTGTCA----- 400
F160_Hi_C             AAAATTGTTGTTGG------CTGTGCGATATAAAAAAAGGTGTCA----- 400
CAMP.105_Hi_C         AAAATTGTTGTTGG------CTGTGCGATATAAAAAAAGGTGTCA----- 400
GELB.122_Hi_C         AAAATTGTTGTTGG------CTGTGCGATATAAAAAAAGGTGTCA----- 400
STGA.116_Hi_C         AAAATTGTTGTTGG------CTGTGCGATATAAAAAAAGGTGTCA----- 400
STRE.219_Hi_C         AAAATTGTTGTTGG------CTGTGCGATATAAAAAAAGGTGTCA----- 400
SATU.189_Hi_C         AAAATTGTTGTTGG------CTGTGCGATATAAAAAAAGGTGTCA----- 400
WALL.165_Hi_C         AAAATTGTTGTTGG------CTGTGCGATATAAAAAAAGGTGTCA----- 400

Maize_B73_RefGen_v4   TGTAAACTGTGAAAAAACTAAAA-TTGTTTGGTGGAAAACCACTAAAAAT 450
L050_Lo_T             TGTAAACTGTGAAAAAACTAAAA-TTGTTTGGTGGAAAACCACTAAAAAT 450
CAMP.107_Lo_T         TGTGAGCTGTGAAAAAACAAAAAGTTGTTTGATGA-AAACCACTAAAAAT 450
GELB.A122_Lo_T        -------------------------------------------------- 450
STGA.104_Lo_T         TGTAAACTGTGAAAAAACTAAAA-TTGTTTGGTGGAAAACCACTAAAAAT 450
SATU.131_Lo_T         TGTAAACTGTGAAAAAACTAAAA-TTGTTTGGTGGAAAACCACTAAAAAT 450
WALL.210_Lo_T         ---------TGAAAAAACTAAAAACTGTTTGGTGG-AAACTACTAAAAAT 450
F169_Lo_C             ---------TGAAAAAACTAAAAACTGTTTGGTGG-AAACTACTAAAAAT 450
STRE.146_Lo_C         ---------TGAAAAAACTAAAAACTGTTTGGTGG-AAACTACTAAAAAT 450
F110_Hi_C             ---------TGAAAAAACTAAAAACTGTTTGGTGG-AAACTACTAAAAAT 450
F160_Hi_C             ---------TGAAAAAACTAAAAACTGTTTGGTGG-AAACTACTAAAAAT 450
CAMP.105_Hi_C         ---------TGAAAAAACTAAAAACTGTTTGGTGG-AAACTACTAAAAAT 450
GELB.122_Hi_C         ---------TGAAAAAACTAAAAACTGTTTGGTGG-AAACTACTAAAAAT 450
STGA.116_Hi_C         ---------TGAAAAAACTAAAAACTGTTTGGTGG-AAACTACTAAAAAT 450
STRE.219_Hi_C         ---------TGAAAAAACTAAAAACTGTTTGGTGG-AAACTACTAAAAAT 450
SATU.189_Hi_C         ---------TGAAAAAACTAAAAACTGTTTGGTGG-AAACTACTAAAAAT 450
WALL.165_Hi_C         ---------TGAAAAAACTAAAAACTGTTTGGTGG-AAACTACTAAAAAT 450

Maize_B73_RefGen_v4   CGTTA-TTTTTTTACAGATATATATTTTTATAGTTCCATCTGAAAGCTAT 500
L050_Lo_T             CGTTA-TTTTTTTACAGATATATATTTTTATAGTTCCATCTGAAAGCTAT 500
CAMP.107_Lo_T         CGTTAAAAGTTTT-TACATATATATTTTCATTGTTGCATCCGAAAGTCAT 500
GELB.A122_Lo_T        -------------------------------------------------- 500
STGA.104_Lo_T         CGTTA-TTTTTTTACAGATATATATTTTTATAGTTCCATCTGAAAGCTAT 500
SATU.131_Lo_T         CGTTA-TTTTTTTACAGATATATATTTTTATAGTTCCATCTGAAAGCTAT 500
WALL.210_Lo_T         CGTTAGTTTTTTTACACACATATATTTTTATAGTTCCATCCGAGAGCTAT 500
F169_Lo_C             CGTTAGTTTTTTTACACACATATATTTTTATAGTTCCATCCGAGAGCTAT 500
STRE.146_Lo_C         CGTTAGTTTTTTTACACACATATATTTTTATAGTTCCATCCGAGAGCTAT 500
F110_Hi_C             CGTTAGTTTTTTTACACACATATATTTTTATAGTTCCATCCGAGAGCTAT 500
F160_Hi_C             CGTTAGTTTTTTTACACACATATATTTTTATAGTTCCATCCGAGAGCTAT 500
CAMP.105_Hi_C         CGTTAGTTTTTTTACACACATATATTTTTATAGTTCCATCCGAGAGCTAT 500
GELB.122_Hi_C         CGTTAGTTTTTTTACACACATATATTTTTATAGTTCCATCCGAGAGCTAT 500
STGA.116_Hi_C         CGTTAGTTTTTTTACACACATATATTTTTATAGTTCCATCCGAGAGCTAT 500
STRE.219_Hi_C         CGTTAGTTTTTTTACACACATATATTTTTATAGTTCCATCCGAGAGCTAT 500
SATU.189_Hi_C         CGTTAGTTTTTTTACACACATATATTTTTATAGTTCCATCCGAGAGCTAT 500
WALL.165_Hi_C         CGTTAGTTTTTTTACACACATATATTTTTATAGTTCCATCCGAGAGCTAT 500
```

```
Maize_B73_RefGen_v4     TAAAAACAGGTCCATAGATGATTTCAATTTTGCACTGCGAGAAGAAAATC 550
L050_Lo_T               TAAAAACAGGTCCATAGATGATTTCAATTTTGCACTGCGAGAAGAAAATC 550
CAMP.107_Lo_T           TAAAAACAGGTACACAAATGATTTTAGTTTTGCACTACG---AGAAAATC 550
GELB.A122_Lo_T          -------------------------------------------------- 550
STGA.104_Lo_T           TAAAAACAGGTCCATAGATGATTTCAATTTTGCACTGCGAGAAGAAAATC 550
SATU.131_Lo_T           TAAAAACAGGTCCATAGATGATTTCAATTTTGCACTGCGAGAAGAAAATC 550
WALL.210_Lo_T           TAAAAACAGGTCCACAGATGATTTC----TTGCACCGCG---AGAAAATC 550
F169_Lo_C               TAAAAACAGGTCCACAGATGATTTC----TTGCACCGCG---AGAAAATC 550
STRE.146_Lo_C           TAAAAACAGGTCCACAGATGATTTC----TTGCACCGCG---AGAAAATC 550
F110_Hi_C               TAAAAACAGGTCCACAGATGATTTC----TTGCATTGCG---AGAAAATC 550
F160_Hi_C               TAAAAACAGGTCCACAGATGATTTC----TTGCATTGCG---AGAAAATC 550
CAMP.105_Hi_C           TAAAAACAGGTCCACAGATGATTTC----TTGCACCGCG---AGAAAATC 550
GELB.122_Hi_C           TAAAAACAGGTCCACAGATGATTTC----TTGCATTGCG---AGAAAATC 550
STGA.116_Hi_C           TAAAAACAGGTCCACAGATGATTTC----TTGCATTGCG---AGAAAATC 550
STRE.219_Hi_C           TAAAAACAGGTCCACAGATGATTTC----TTGCATTGCG---AGAAAATC 550
SATU.189_Hi_C           TAAAAACAGGTCCACAGATGATTTC----TTGCACCGCG---AGAAAATC 550
WALL.165_Hi_C           TAAAAACAGGTCCACAGATGATTTC----TTGCACCGCG---AGAAAATC 550

Maize_B73_RefGen_v4     GTCTTTTAGAAGGAAAAAAATGCTTTCTAAATTCAACCTTTTGATTTATA 600
L050_Lo_T               GTCTTTTAGAAGGAAAAAAATGCTTTCTAAATTCAACCTTTTGATTTATA 600
CAMP.107_Lo_T           GTCTTTTATGAAAAAAAAA-TGCTTTCTGAATCTAACTCTTTGGTTTAGG 600
GELB.A122_Lo_T          -------------------------------------------------- 600
STGA.104_Lo_T           GTCTTTTAGAAGGAAAAAAATGCTTTCTAAATTCAACCTTTTGATTTATA 600
SATU.131_Lo_T           GTCTTTTAGAAGGAAAAAAATGCTTTCTAAATTCAACCTTTTGATTTATA 600
WALL.210_Lo_T           GTCTTTTAGAAAGAAAAAACTGCTTTCTGAATTCAACCTTTTAATTTATA 600
F169_Lo_C               GTCTTTTAGAAAGAAAAAACTGCTTTCTGAATTCAACCTTTTAATTTATA 600
STRE.146_Lo_C           GTCTTTTAGAAAGAAAAAACTGCTTTCTGAATTCAACCTTTTAATTTATA 600
F110_Hi_C               GTCTTTTAGAAAGAAAAAACTGCTTTCTGAATTCAACCTTTTAATTTATA 600
F160_Hi_C               GTCTTTTAGAAAGAAAAAACTGCTTTCTGAATTCAACCTTTTAATTTATA 600
CAMP.105_Hi_C           GTCTTTTAGAAAGAAAAAACTGCTTTCTGAATTCAACCTTTTAATTTATA 600
GELB.122_Hi_C           GTCTTTTAGAAAGAAAAAACTGCTTTCTGAATTCAACCTTTTAATTTATA 600
STGA.116_Hi_C           GTCTTTTAGAAAGAAAAAACTGCTTTCTGAATTCAACCTTTTAATTTATA 600
STRE.219_Hi_C           GTCTTTTAGAAAGAAAAAACTGCTTTCTGAATTCAACCTTTTAATTTATA 600
SATU.189_Hi_C           GTCTTTTAGAAAGAAAAAACTGCTTTCTGAATTCAACCTTTTAATTTATA 600
WALL.165_Hi_C           GTCTTTTAGAAAGAAAAAACTGCTTTCTGAATTCAACCTTTTAATTTATA 600

Maize_B73_RefGen_v4     TTTTATTTTTAGGGACAAAAACTAATGCCA-AAGATAAACCAAATACACC 650
L050_Lo_T               TTTTATTTTTAGGGACAAAAACTAATGCCA-AAGATAAACCAAATACACC 650
CAMP.107_Lo_T           TTTTGTTTTTTTAGATAAAACTAAAGAC---AAGATAAACCAAACACACC 650
GELB.A122_Lo_T          -------------------------------------------------- 650
STGA.104_Lo_T           TTTTATTTTTAGGGACAAAAACTAATGCCA-AAGATAAACCAAATACACC 650
SATU.131_Lo_T           TTTTATTTTTAGGGACAAAAACTAATGCCA-AAGATAAACCAAATACACC 650
WALL.210_Lo_T           TTTTATTTTTAGGGACAAAAACTAATGCCAAAAGATAAACCAAATACACC 650
F169_Lo_C               TTTTATTTTTAGGGACAAAAACTAATGCCAAAAGATAAACCAAATACACC 650
STRE.146_Lo_C           TTTTATTTTTAGGGACAAAAACTAATGCCAAAAGATAAACCAAATACACC 650
F110_Hi_C               TTTTATTTTTAGGGACAAAAACTAATGCCAAAAGATAAACCAAATACACC 650
F160_Hi_C               TTTTATTTTTAGGGACAAAAACTAATGCCAAAAGATAAACCAAATACACC 650
CAMP.105_Hi_C           TTTTATTTTTAGGGACAAAAACTAATGCCAAAAGATAAACCAAATACACC 650
GELB.122_Hi_C           TTTTATTTTTAGGGACAAAAACTAATGCCAAAAGATAAACCAAATACACC 650
STGA.116_Hi_C           TTTTATTTTTAGGGACAAAAACTAATGCCAAAAGATAAACCAAATACACC 650
STRE.219_Hi_C           TTTTATTTTTAGGGACAAAAACTAATGCCAAAAGATAAACCAAATACACC 650
SATU.189_Hi_C           TTTTATTTTTAGGGACAAAAACTAATGCCAAAAGATAAACCAAATACACC 650
WALL.165_Hi_C           TTTTATTTTTAGGGACAAAAACTAATGCCAAAAGATAAACCAAATACACC 650

Maize_B73_RefGen_v4     GAATTTCCGATCTGAACTT-GTGCCAAGCGGTGGTCGAAATCCATCGCAG 700
L050_Lo_T               GAATTTCCGATCTGAACTT-GTGCCAAGCGGTGGTCGAAATCCATCGCAG 700
CAMP.107_Lo_T           GAATTTCCGATCTGAACTTAGTGCCAAGCGGTGGTCGAAATCCACCGCAG 700
GELB.A122_Lo_T          -------------------------------------------------- 700
STGA.104_Lo_T           GAATTTCCGATCTGAACTT-GTGCCAAGCGGTGGTCGAAATCCATCGCAG 700
SATU.131_Lo_T           GAATTTCCGATCTGAACTT-GTGCCAAGCGGTGGTCGAAATCCATCGCAG 700
WALL.210_Lo_T           GAA-TTCCGACCTGAACTT-GTGCCAAGCGGTGGTCGAAATCCATCGCAG 700
F169_Lo_C               GAA-TTCCGACCTGAACTT-GTGCCAAGCGGTGGTCGAAATCCATCGCAG 700
STRE.146_Lo_C           GAA-TTCCGACCTGAACTT-GTGCCAAGCGGTGGTCGAAATCCATCGCAG 700
F110_Hi_C               GAA-TTCCGACCTGAACTT-GTGCCAAGCGGTGGTCGAAATCCATCGCAG 700
```

```
F160_Hi_C            GAA-TTCCGACCTGAACTT-GTGCCAAGCGGTGGTCGAAATCCATCGCAG 700
CAMP.105_Hi_C        GAA-TTCCGACCTGAACTT-GTGCCAAGCGGTGGTCGAAATCCATCGCAG 700
GELB.122_Hi_C        GAA-TTCCGACCTGAACTT-GTGCCAAGCGGTGGTCGAAATCCATCGCAG 700
STGA.116_Hi_C        GAA-TTCCGACCTGAACTT-GTGCCAAGCGGTGGTCGAAATCCATCGCAG 700
STRE.219_Hi_C        GAA-TTCCGACCTGAACTT-GTGCCAAGCGGTGGTCGAAATCCATCGCAG 700
SATU.189_Hi_C        GAA-TTCCGACCTGAACTT-GTGCCAAGCGGTGGTCGAAATCCATCGCAG 700
WALL.165_Hi_C        GAA-TTCCGACCTGAACTT-GTGCCAAGCGGTGGTCGAAATCCATCGCAG 700

Maize_B73_RefGen_v4  CTAGCTTATGAAGAGGACAAGGCTTTGGACTAATCAATCATGCGCGTGCA 750
L050_Lo_T            CTAGCTTATGAAGAGGACAAGGCTTTGGACTAATCAATCATGCGCGTGCA 750
CAMP.107_Lo_T        CTAGCTTATGAAGAGGACAAGGCTTTGGACTAATCAATCATGCGCGTGCA 750
GELB.A122_Lo_T       -------------------------------------------------- 750
STGA.104_Lo_T        CTAGCTTATGAAGAGGACAAGGCTTTGGACTAATCAATCATGCGCGTGCA 750
SATU.131_Lo_T        CTAGCTTATGAAGAGGACAAGGCTTTGGACTAATCAATCATGCGCGTGCA 750
WALL.210_Lo_T        CTAGCTTATGAAGAGGACAAGGCTTTGGACTAATCAATCATGCGCGTGCA 750
F169_Lo_C            CTAGCTTATGAAGAGGACAAGGCTTTGGACTAATCAATCATGCGCGTGCA 750
STRE.146_Lo_C        CTAGCTTATGAAGAGGACAAGGCTTTGGACTAATCAATCATGCGCGTGCA 750
F110_Hi_C            CTAGCTTATGAAGAGGACAAGGCTTTGGACTAATCAATCATGCGCGTGCA 750
F160_Hi_C            CTAGCTTATGAAGAGGACAAGGCTTTGGACTAATCAATCATGCGCGTGCA 750
CAMP.105_Hi_C        CTAGCTTATGAAGAGGACAAGGCTTTGGACTAATCAATCATGCGCGTGCA 750
GELB.122_Hi_C        CTAGCTTATGAAGAGGACAAGGCTTTGGACTAATCAATCATGCGCGTGCA 750
STGA.116_Hi_C        CTAGCTTATGAAGAGGACAAGGCTTTGGACTAATCAATCATGCGCGTGCA 750
STRE.219_Hi_C        CTAGCTTATGAAGAGGACAAGGCTTTGGACTAATCAATCATGCGCGTGCA 750
SATU.189_Hi_C        CTAGCTTATGAAGAGGACAAGGCTTTGGACTAATCAATCATGCGCGTGCA 750
WALL.165_Hi_C        CTAGCTTATGAAGAGGACAAGGCTTTGGACTAATCAATCATGCGCGTGCA 750

Maize_B73_RefGen_v4  ATTGCACGGTTTGTGGCTGTAATTTAATCTCTGCATAGAGCGTAGAGTGC 800
L050_Lo_T            ATTGCACGGTTTGTGGCTGTAATTTAATCTCTGCATAGAGCGTAGAGTGC 800
CAMP.107_Lo_T        ATTGCACGGTTTGTGGCTGTAATTTAATCTCTGCATAGAGCGTAGAGTGC 800
GELB.A122_Lo_T       -------------------------------------------------- 800
STGA.104_Lo_T        ATTGCACGGTTTGTGGCTGTAATTTAATCTCTGCATAGAGCGTAGAGTGC 800
SATU.131_Lo_T        ATTGCACGGTTTGTGGCTGTAATTTAATCTCTGCATAGAGCGTAGAGTGC 800
WALL.210_Lo_T        ATTGCACGGTTTGTGGCTGTAATTTAATCTCTGCATAGAGCGTAGAGTGC 800
F169_Lo_C            ATTGCACGGTTTGTGGCTGTAATTTAATCTCTGCATAGAGCGTAGAGTGC 800
STRE.146_Lo_C        ATTGCACGGTTTGTGGCTGTAATTTAATCTCTGCATAGAGCGTAGAGTGC 800
F110_Hi_C            ATTGCACGGTTTGTGGCTGTAATTTAATCTCTGCATAGAGCGTAGAGTGC 800
F160_Hi_C            ATTGCACGGTTTGTGGCTGTAATTTAATCTCTGCATAGAGCGTAGAGTGC 800
CAMP.105_Hi_C        ATTGCACGGTTTGTGGCTGTAATTTAATCTCTGCATAGAGCGTAGAGTGC 800
GELB.122_Hi_C        ATTGCACGGTTTGTGGCTGTAATTTAATCTCTGCATAGAGCGTAGAGTGC 800
STGA.116_Hi_C        ATTGCACGGTTTGTGGCTGTAATTTAATCTCTGCATAGAGCGTAGAGTGC 800
STRE.219_Hi_C        ATTGCACGGTTTGTGGCTGTAATTTAATCTCTGCATAGAGCGTAGAGTGC 800
SATU.189_Hi_C        ATTGCACGGTTTGTGGCTGTAATTTAATCTCTGCATAGAGCGTAGAGTGC 800
WALL.165_Hi_C        ATTGCACGGTTTGTGGCTGTAATTTAATCTCTGCATAGAGCGTAGAGTGC 800

Maize_B73_RefGen_v4  AATCCGCATCAAACGCCGGTTGGGAGCGGGAGCAGCCAATGGAAACCCTC 850
L050_Lo_T            AATCCGCATCAAACGCCGGTTGGGAGCGGGAGCAGCCAATGGAAACCCTC 850
CAMP.107_Lo_T        AATCCGCATCAAACGCCGGTTGGGAGCGGGAGCAGCCAATGGAAACCCTC 850
GELB.A122_Lo_T       --------------------------------------------ACCCTC 850
STGA.104_Lo_T        AATCCGCATCAAACGCCGGTTGGGAGCGGGAGCAGCCAATGGAAACCCTC 850
SATU.131_Lo_T        AATCCGCATCAAACGCCGGTTGGGAGCGGGAGCAGCCAATGGAAACCCTC 850
WALL.210_Lo_T        AATCCGCATCAAACGCCGGTTGGGAGCGGGAGCAGCCAATGGAGACCCTC 850
F169_Lo_C            AATCCGCATCAAACGCCGGTTGGGAGCGGGAGCAGCCAATGGAGACCCTC 850
STRE.146_Lo_C        AATCCGCATCAAACGCCGGTTGGGAGCGGGAGCAGCCAATGGAGACCCTC 850
F110_Hi_C            AATCCGCATCAAACGCCGGTTGGGAGCGGGAGCAGCCAATGGAGACCCTC 850
F160_Hi_C            AATCCGCATCAAACGCCGGTTGGGAGCGGGAGCAGCCAATGGAGACCCTC 850
CAMP.105_Hi_C        AATCCGCATCAAACGCCGGTTGGGAGCGGGAGCAGCCAATGGAGACCCTC 850
GELB.122_Hi_C        AATCCGCATCAAACGCCGGTTGGGAGCGGGAGCAGCCAATGGAGACCCTC 850
STGA.116_Hi_C        AATCCGCATCAAACGCCGGTTGGGAGCGGGAGCAGCCAATGGAGACCCTC 850
STRE.219_Hi_C        AATCCGCATCAAACGCCGGTTGGGAGCGGGAGCAGCCAATGGAGACCCTC 850
SATU.189_Hi_C        AATCCGCATCAAACGCCGGTTGGGAGCGGGAGCAGCCAATGGAGACCCTC 850
WALL.165_Hi_C        AATCCGCATCAAACGCCGGTTGGGAGCGGGAGCAGCCAATGGAGACCCTC 850

Maize_B73_RefGen_v4  CGAAATCGAGAGCCGCGT--CACACACACACACTCTCCCACGGTGCCCTG 900
L050_Lo_T            CGAAATCGAGAGCCGCGT--CACACACACACACTCTCCCACGGTGCCCTG 900
```

```
CAMP.107_Lo_T         CGAAATCGAGAGCCGCGTCACACACACACACACTCTCCCACGGTGCCCTG 900
GELB.A122_Lo_T        CGAAATCGAGAGCCGCGT--CACACACACACACTCTCCCACGGTGCCCTG 900
STGA.104_Lo_T         CGAAATCGAGAGCCGCGT--CACACACACACACTCTCCCACGGTGCCCTG 900
SATU.131_Lo_T         CGAAATCGAGAGCCGCGT--CACACACACACACTCTCCCACGGTGCCCTG 900
WALL.210_Lo_T         CGAAATCGAGAGCCGCGT------CACACACACTCTCCCACGTTGCCCTG 900
F169_Lo_C             CGAAATCGAGAGCCGCGT------CACACACACTCTCCCACGTTGCCCTG 900
STRE.146_Lo_C         CGAAATCGAGAGCCGCGT------CACACACACTCTCCCACGTTGCCCTG 900
F110_Hi_C             CGAAATCGAGAGCCGCGT------CACACACACTCTCCCACGTTGCCCTG 900
F160_Hi_C             CGAAATCGAGAGCCGCGT------CACACACACTCTCCCACGTTGCCCTG 900
CAMP.105_Hi_C         CGAAATCGAGAGCCGCGT------CACACACACTCTCCCACGTTGCCCTG 900
GELB.122_Hi_C         CGAAATCGAGAGCCGCGT------CACACACACTCTCCCACGTTGCCCTG 900
STGA.116_Hi_C         CGAAATCGAGAGCCGCGT------CACACACACTCTCCCACGTTGCCCTG 900
STRE.219_Hi_C         CGAAATCGAGAGCCGCGT------CACACACACTCTCCCACGTTGCCCTG 900
SATU.189_Hi_C         CGAAATCGAGAGCCGCGT------CACACACACTCTCCCACGTTGCCCTG 900
WALL.165_Hi_C         CGAAATCGAGAGCCGCGT------CACACACACTCTCCCACGTTGCCCTG 900

                                    KASP
Maize_B73_RefGen_v4   CAAATAAAAAAAAATCAAGAATCTCGGATTTTCGGCTTCCTCAAAAGCCC 950
L050_Lo_T             CAAATAAAAAAAAATCAAGAATCTCGGATTTTCGGCTTCCTCAAAAGCCC 950
CAMP.107_Lo_T         CAAATAAAAAAAAATCAAGAATCTCGGATTTTCGGCTTCCTCAAAAGCCC 950
GELB.A122_Lo_T        CAAATAAAAAAAAA-CAAGAATCTCGGATTTTCGGCTTCCTCAAAAGCCC 950
STGA.104_Lo_T         CAAATAAAAAAAAATCAAGAATCTCGGATTTTCGGCTTCCTCAAAAGCCC 950
SATU.131_Lo_T         CAAATAAAAAAAAATCAAGAATCTCGGATTTTCGGCTTCCTCAAAAGCCC 950
WALL.210_Lo_T         CAAATAAAAAAA-------------------------------------- 950
F169_Lo_C             CAAATAAAAAAAAA---AGAATCTCGGATTTTCGGCTTCCTCAAAAGCCC 950
STRE.146_Lo_C         CAAATAAAAAAAAA---AGAATCTCGGATTTTCGGCTTCCTCAAAAGCCC 950
F110_Hi_C             CAAATAAAAAAAAA----GAATCTCGGATTTTCGGCTTCCTCAAAAGCCC 950
F160_Hi_C             CAAATAAAAAAAAA----GAATCTCGGATTTTCGGCTTCCTCAAAAGCCC 950
CAMP.105_Hi_C         CAAATAAAAAAAAA---AGAATCTCGGATTTTCGGCTTCCTCAAAAGCCC 950
GELB.122_Hi_C         CAAATAAAAAAAAA----GAATCTCGGATTTTCGGCTTCCTCAAAAGCCC 950
STGA.116_Hi_C         CAAATAAAAAAAAA----GAATCTCGGATTTTCGGCTTCCTCAAAAGCCC 950
STRE.219_Hi_C         CAAATAAAAAAAAA----GAATCTCGGATTTTCGGCTTCCTCAAAAGCCC 950
SATU.189_Hi_C         CAAATAAAAAAAAA---AGAATCTCGGATTTTCGGCTTCCTCAAAAGCCC 950
WALL.165_Hi_C         CAAATAAAAAAAAA---AGAATCTCGGATTTTCGGCTTCCTCAAAAGCCC 950


Maize_B73_RefGen_v4   CCAGAGATTCCCAAAAGGTTTAAGCCACTTGGATGGCGACCGCACACAAC 1000
L050_Lo_T             CCAGAGATTCCCAAAAGGTTTAAGCCACTTGGATGGCGACCGCACACAAC 1000
CAMP.107_Lo_T         CCAGAGATTCCCAAAAGGTTTAAGCCACTTGGATGGCGACCGCACACAAC 1000
GELB.A122_Lo_T        CCAGAGATTCCCAAAAGGTTTAAGCCACTTGGATGGCGACCGCACACAAC 1000
STGA.104_Lo_T         CCAGAGATTCCCAAAAGGTTTAAGCCACTTGGATGGCGACCGCACACAAC 1000
SATU.131_Lo_T         CCAGAGATTCCCAAAAGGTTTAAGCCACTTGGATGGCGACCGCACACAAC 1000
WALL.210_Lo_T         -------------------------------------------------- 1000
F169_Lo_C             CCAGAGATTCCCAAAAGGTTTAAGCCACTTGGATGGCGACCGCACACAAC 1000
STRE.146_Lo_C         CCAGAGATTCCCAAAAGGTTTAAGCCACTTGGATGGCGACCGCACACAAC 1000
F110_Hi_C             CCAGAGATTCCCAAAAGGTTTAAGCCACTTGGATGGCGACCGCACACAAC 1000
F160_Hi_C             CCAGAGATTCCCAAAAGGTTTAAGCCACTTGGATGGCGACCGCACACAAC 1000
CAMP.105_Hi_C         CCAGAGATTCCCAAAAGGTTTAAGCCACTTGGATGGCGACCGCACACAAC 1000
GELB.122_Hi_C         CCAGAGATTCCCAAAAGGTTTAAGCCACTTGGATGGCGACCGCACACAAC 1000
STGA.116_Hi_C         CCAGAGATTCCCAAAAGGTTTAAGCCACTTGGATGGCGACCGCACACAAC 1000
STRE.219_Hi_C         CCAGAGATTCCCAAAAGGTTTAAGCCACTTGGATGGCGACCGCACACAAC 1000
SATU.189_Hi_C         CCAGAGATTCCCAAAAGGTTTAAGCCACTTGGATGGCGACCGCACACAAC 1000
WALL.165_Hi_C         CCAGAGATTCCCAAAAGGTTTAAGCCACTTGGATGGCGACCGCACACAAC 1000


Maize_B73_RefGen_v4   GTTACAGGACCAGCTTGGCGCACACCCGGTCCAGTGGACCCTGACACCGT 1050
L050_Lo_T             GTTACAGGACCAGCTTGGCGCACACCCGGTCCAGTGGACCCTGACACCGT 1050
CAMP.107_Lo_T         GTTACAGGACCAGCTTGGCGCACACCCGGTCCAGTGGACCCTGACACCGT 1050
GELB.A122_Lo_T        GTTACAGGACCAGCTTGGCGCACACCCGGTCCAGTGGACCCTGACACCGC 1050
STGA.104_Lo_T         GTTACAGGACCAGCTTGGCGCACACCCGGTCCAGTGGACCCTGACACCGT 1050
SATU.131_Lo_T         GTTACAGGACCAGCTTGGCGCACACCCGGTCCAGTGGACCCTGACACCGT 1050
WALL.210_Lo_T         -------------------------------------------------- 1050
F169_Lo_C             GTTACAGGACCAGCTTGGCGCACACCCGGTCCAGTGGACCCTGACACCGT 1050
STRE.146_Lo_C         GTTACAGGACCAGCTTGGCGCACACCCGGTCCAGTGGACCCTGACACCGT 1050
F110_Hi_C             GTTACAGGACCAGCTTGGCGCACACCCGGTCCAGTGGACCCTGACACCGT 1050
F160_Hi_C             GTTACAGGACCAGCTTGGCGCACACCCGGTCCAGTGGACCCTGACACCGT 1050
CAMP.105_Hi_C         GTTACAGGACCAGCTTGGCGCACACCCGGTCCAGTGGACCCTGACACCGT 1050
GELB.122_Hi_C         GTTACAGGACCAGCTTGGCGCACACCCGGTCCAGTGGACCCTGACACCGT 1050
```

```
STGA.116_Hi_C          GTTACAGGACCAGCTTGGCGCACACCCGGTCCAGTGGACCCTGACACCGT 1050
STRE.219_Hi_C          GTTACAGGACCAGCTTGGCGCACACCCGGTCCAGTGGACCCTGACACCGT 1050
SATU.189_Hi_C          GTTACAGGACCAGCTTGGCGCACACCCGGTCCAGTGGACCCTGACACCGT 1050
WALL.165_Hi_C          GTTACAGGACCAGCTTGGCGCACACCCGGTCCAGTGGACCCTGACACCGT 1050


Maize_B73_RefGen_v4    ACATACCACCGGCATCCGGGGCCCGCAGGAATCCACGAGCCCACGGGTTG 1100
L050_Lo_T              ACATACCACCGGCATCCGGGGCCCGCAGGAATCCACGAGCCCACGGGTTG 1100
CAMP.107_Lo_T          ACATACCACCGGCATCCGGGGCCCGCAGGAATCCACGAGCCCACGGGTTG 1100
GELB.A122_Lo_T         ACATACCACCGGCATCCAGGGCCCGCAGGAATCCACGAGCCCACGGGTTG 1100
STGA.104_Lo_T          ACATACCACCGGCATCCGGGGCCCGCAGGAATCCACGAGCCCACGGGTTG 1100
SATU.131_Lo_T          ACATACCACCGGCATCCGGGGCCCGCAGGAATCCACGAGCCCACGGGTTG 1100
WALL.210_Lo_T          -------------------------------------------------- 1100
F169_Lo_C              ACATACCACCGGCATCCGGGGCCCGCAGGAATCCACGAGCCCACGGGTTG 1100
STRE.146_Lo_C          ACATACCACCGGCATCCGGGGCCCGCAGGAATCCACGAGCCCACGGGTTG 1100
F110_Hi_C              ACATACCACCGGCATCCGGGGCCCGCAGGAATCCACGAGCCCACGGGTTG 1100
F160_Hi_C              ACATACCACCGGCATCCGGGGCCCGCAGGAATCCACGAGCCCACGGGTTG 1100
CAMP.105_Hi_C          ACATACCACCGGCATCCGGGGCCCGCAGGAATCCACGAGCCCACGGGTTG 1100
GELB.122_Hi_C          ACATACCACCGGCATCCGGGGCCCGCAGGAATCCACGAGCCCACGGGTTG 1100
STGA.116_Hi_C          ACATACCACCGGCATCCGGGGCCCGCAGGAATCCACGAGCCCACGGGTTG 1100
STRE.219_Hi_C          ACATACCACCGGCATCCGGGGCCCGCAGGAATCCACGAGCCCACGGGTTG 1100
SATU.189_Hi_C          ACATACCACCGGCATCCGGGGCCCGCAGGAATCCACGAGCCCACGGGTTG 1100
WALL.165_Hi_C          ACATACCACCGGCATCCGGGGCCCGCAGGAATCCACGAGCCCACGGGTTG 1100


Maize_B73_RefGen_v4    GGTGATCCGGATCCACGCGACCACCTCCACCAAGCCACGAGTAGACTGAG 1150
L050_Lo_T              GGTGATCCGGATCCACGCGACCACCTCCACCAAGCCACGAGTAGACTGAG 1150
CAMP.107_Lo_T          GGTGATCCGGATCCACGCGACCACCTCCACCAAGCCACGAGTAGACTGAG 1150
GELB.A122_Lo_T         GGTGCTCCGGATCCACGCGACCACCTCCACCAAGCCACGAGTAGACTGAG 1150
STGA.104_Lo_T          GGTGATCCGGATCCACGCGACCACCTCCACCAAGCCACGAGTAGACTGAG 1150
SATU.131_Lo_T          GGTGATCCGGATCCACGCGACCACCTCCACCAAGCCACGAGTAGACTGAG 1150
WALL.210_Lo_T          -------------------------------------------------- 1150
F169_Lo_C              GGTGATCCGGATCCACGCGACCACCTCCACCAAGCCACGAGTAGACTGAG 1150
STRE.146_Lo_C          GGTGATCCGGATCCACGCGACCACCTCCACCAAGCCACGAGTAGACTGAG 1150
F110_Hi_C              GGTGATCCGGATCCACGCGACCACCTCCACCAAGCCACGAGTAGACTGAG 1150
F160_Hi_C              GGTGATCCGGATCCACGCGACCACCTCCACCAAGCCACGAGTAGACTGAG 1150
CAMP.105_Hi_C          GGTGATCCGGATCCACGCGACCACCTCCACCAAGCCACGAGTAGACTGAG 1150
GELB.122_Hi_C          GGTGATCCGGATCCACGCGACCACCTCCACCAAGCCACGAGTAGACTGAG 1150
STGA.116_Hi_C          GGTGATCCGGATCCACGCGACCACCTCCACCAAGCCACGAGTAGACTGAG 1150
STRE.219_Hi_C          GGTGATCCGGATCCACGCGACCACCTCCACCAAGCCACGAGTAGACTGAG 1150
SATU.189_Hi_C          GGTGATCCGGATCCACGCGACCACCTCCACCAAGCCACGAGTAGACTGAG 1150
WALL.165_Hi_C          GGTGATCCGGATCCACGCGACCACCTCCACCAAGCCACGAGTAGACTGAG 1150


Maize_B73_RefGen_v4    CGCGGGATCCCACGGGCAGAAAGGGACGCGACTCGTCAGGGCGATTCCGA 1200
L050_Lo_T              CGCGGGATCCCACGGGCAGAAAGGGACGCGACTCGTCAGGGCGATTCCGA 1200
CAMP.107_Lo_T          CGCGGGATCCCACGGGCAGAAAGGGACGCGACTCGTCAGGGCGATTCCGA 1200
GELB.A122_Lo_T         CGCGGGATCCCACGGGCAGAAAGGGACGCGACTCGTCAGGGCGATTCCGA 1200
STGA.104_Lo_T          CGCGGGATCCCACGGGCAGAAAGGGACGCGACTCGTCAGGGCGATTCCGA 1200
SATU.131_Lo_T          CGCGGGATCCCACGGGCAGAAAGGGACGCGACTCGTCAGGGCGATTCCGA 1200
WALL.210_Lo_T          -------------------------------------------------- 1200
F169_Lo_C              CGCGGGATCCCACGGGCAGAAAGGGACGCGACTCGTCAGGGCGATTCCGA 1200
STRE.146_Lo_C          CGCGGGATCCCACGGGCAGAAAGGGACGCGACTCGTCAGGGCGATTCCGA 1200
F110_Hi_C              CGCGGGATCCCACGGGCAGAAAGGGACGCGACTCGTCAGGGCGATTCCGA 1200
F160_Hi_C              CGCGGGATCCCACGGGCAGAAAGGGACGCGACTCGTCAGGGCGATTCCGA 1200
CAMP.105_Hi_C          CGCGGGATCCCACGGGCAGAAAGGGACGCGACTCGTCAGGGCGATTCCGA 1200
GELB.122_Hi_C          CGCGGGATCCCACGGGCAGAAAGGGACGCGACTCGTCAGGGCGATTCCGA 1200
STGA.116_Hi_C          CGCGGGATCCCACGGGCAGAAAGGGACGCGACTCGTCAGGGCGATTCCGA 1200
STRE.219_Hi_C          CGCGGGATCCCACGGGCAGAAAGGGACGCGACTCGTCAGGGCGATTCCGA 1200
SATU.189_Hi_C          CGCGGGATCCCACGGGCAGAAAGGGACGCGACTCGTCAGGGCGATTCCGA 1200
WALL.165_Hi_C          CGCGGGATCCCACGGGCAGAAAGGGACGCGACTCGTCAGGGCGATTCCGA 1200


Maize_B73_RefGen_v4    TTACGAGTAGACGGGCAGGACAGGACAAGGGAGGAA---CGGCGGCGGCG 1250
L050_Lo_T              TTACGAGTAGACGGGCAGGACAGGACAAGGGAGGAA---CGGCGGCGGCG 1250
CAMP.107_Lo_T          CTACGAGGAGACGGGCAGGACAGGACAAGGGAGGAACGGCGGCGGCGGCG 1250
GELB.A122_Lo_T         CTTCGAGGAGACGGGCAGGACAGGACAAGGGAGGAA------CGGCGGCG 1250
STGA.104_Lo_T          TTACGAGTAGACGGGCAGGACAGGACAAGGGAGGAA---CGGCGGCGGCG 1250
```

```
SATU.131_Lo_T        TTACGAGTAGACGGGCAGGACAGGACAAGGGAGGAA---CGGCGGCGGCG 1250
WALL.210_Lo_T        -------------------------------------------------- 1250
F169_Lo_C            TTACGAGTAGACGGGCAGGACAGGACAAGGGAGGAA---CGGCGGCGGCG 1250
STRE.146_Lo_C        TTACGAGTAGACGGGCAGGACAGGACAAGGGAGGAA---CGGCGGCGGCG 1250
F110_Hi_C            TTACGAGTAGACGGGCAGGACAGGACAAGGGAGGAA---CGGCGGCGGCG 1250
F160_Hi_C            TTACGAGTAGACGGGCAGGACAGGACAAGGGAGGAA---CGGCGGCGGCG 1250
CAMP.105_Hi_C        TTACGAGTAGACGGGCAGGACAGGACAAGGGAGGAA---CGGCGGCGGCG 1250
GELB.122_Hi_C        TTACGAGTAGACGGGCAGGACAGGACAAGGGAGGAA---CGGCGGCGGCG 1250
STGA.116_Hi_C        TTACGAGTAGACGGGCAGGACAGGACAAGGGAGGAA---CGGCGGCGGCG 1250
STRE.219_Hi_C        TTACGAGTAGACGGGCAGGACAGGACAAGGGAGGAA---CGGCGGCGGCG 1250
SATU.189_Hi_C        TTACGAGTAGACGGGCAGGACAGGACAAGGGAGGAA---CGGCGGCGGCG 1250
WALL.165_Hi_C        TTACGAGTAGACGGGCAGGACAGGACAAGGGAGGAA---CGGCGGCGGCG 1250

Maize_B73_RefGen_v4  GCGCAGGGCAGGGTAAACCATG 1272
L050_Lo_T            GCGCAGGGCAGGGTAAACCATG 1272
CAMP.107_Lo_T        GCGCAGGGCAGGGTAAACCATG 1272
GELB.A122_Lo_T       GCGCAGGGCAGGGTAAACCATG 1272
STGA.104_Lo_T        GCGCAGGGCAGGGTAAACCATG 1272
SATU.131_Lo_T        GCGCAGGGCAGGGTAAACCATG 1272
WALL.210_Lo_T        ---------------------- 1272
F169_Lo_C            GCGCAGGGCAGGGTAAACCATG 1272
STRE.146_Lo_C        GCGCAGGGCAGGGTAAACCATG 1272
F110_Hi_C            GCGCAAGGCAGGGTAAACCATG 1272
F160_Hi_C            GCGCAGGGCAGGGTAAACCATG 1272
CAMP.105_Hi_C        GCGCAGGGCAGGGTAAACCATG 1272
GELB.122_Hi_C        GCGCAGGGCAGGGTAAACCATG 1272
STGA.116_Hi_C        GCGCAGGGCAGGGTAAACCATG 1272
STRE.219_Hi_C        GCGCAGGGCAGGGTAAACCATG 1272
SATU.189_Hi_C        GCGCAGGGCAGGGTAAACCATG 1272
WALL.165_Hi_C        GCGCAGGGCAGGGTAAACCATG 1272
```

Table S7 Effects of the identified polymorphisms in the allantoinase gene and its promoter. Proportion of explained genotypic variance (π_G in %) and additive effect (a-Effect) of the QTL in the panel of landraces and in each landrace are shown based on genotyping with the developed KASP markers. In addition, results are shown for marker AX-90560856 from the 50k SNP array. *P* values are derived from the association mixed model analysis with a kinship matrix and the fixed effect for the population.

	AX-90560856	-350 (TCA/---)	C721A (L107M)	G3615A (D454N)
Entire landrace panel				
P value	5.20e-6	1.79e-6	1.69e-3	0.65
Freq.	0.63	0.77	0.54	0.62
π_G	8.2	18.7	2.5	0.1
a-Effect	0.16	0.28	0.09	-0.01
Within landraces and elite lines				
Freq. in CG	0.58	0.61	0.00	0.58
Freq. in GB	0.98	0.98	0.98	0.66
Freq. in RT	0.94	0.94	0.94	0.87
Freq. in SF	1.00	1.00	1.00	0.90
Freq. in SM	0.35	0.86	0.19	0.34
Freq. in WA	0.42	0.42	0.41	0.68
Freq. in EF	0.98	0.98	0.98	0.98
π_G in CG	37.1	19.8	-	37.1
π_G in GB	0.6	0.6	0.6	9.4
π_G in RT	5.1	4.4	4.4	7.3
π_G in SF	-	-	-	26.0
π_G in SM	0.1	0.4	3.9	0.2
π_G in WA	39.9	36.3	35.7	20.5
π_G in EF	6.5	6.1	6.2	7.3
a-Effect in CG	0.28	0.20	-	0.28
a-Effect in GB	0.14	0.14	0.14	0.16
a-Effect in RT	0.25	0.23	0.23	0.21
a-Effect in SF	-	-	-	0.40
a-Effect in SM	-0.02	-0.04	-0.12	-0.02
a-Effect in WA	0.30	0.29	0.29	-0.23
a-Effect in EF	0.35	0.35	0.35	0.35

CG, Campan Galade; GB, Gelber Badischer; RT, Rheintaler; SF, Strenzfelder; SM, Satu Mare; WA, Walliser; EF, elite Flint. Freq., π_G and a-Effect refer to the allele 'C' for marker AX-90560856 [C/T], the deletion for the promoter InDel at -350, allele 'A' for C721A, and allele 'A' for G3615A.

Table S8 Promoter InDel and allantoin content in the landrace 'Walliser'.

Genotype	Promoter InDel	Allantoin content
WALL.296	InsIns	7.7360
WALL.175	InsIns	7.7409
WALL.254	InsIns	7.7497
WALL.277	InsIns	7.7607
WALL.197	InsIns	7.7638
WALL.207	InsIns	7.7720
WALL.219	InsIns	7.7921
WALL.001	InsIns	7.7940
WALL.169	InsIns	7.7955
WALL.257	InsIns	7.8303
WALL.228	InsIns	7.9083
WALL.168	InsIns	7.9092
WALL.105	InsIns	7.9315
WALL.128	InsIns	7.9718
WALL.215	InsIns	7.9856
WALL.014	InsIns	7.9891
WALL.270	InsIns	7.9913
WALL.122	InsIns	8.0000
WALL.134	InsIns	8.0023
WALL.113	InsIns	8.0043
WALL.301	InsIns	8.0138
WALL.269	InsIns	8.0252
WALL.185	InsIns	8.0791
WALL.191	DelDel	8.2658
WALL.154	InsIns	8.2669
WALL.285	DelDel	8.2903
WALL.104	NA	8.3267
WALL.309	InsIns	8.3300
WALL.195	DelDel	8.3821
WALL.222	DelDel	8.4071
WALL.184	InsIns	8.4551
WALL.012	InsIns	8.4710
WALL.178	DelDel	8.5204
WALL.213	DelDel	8.5758
WALL.174	DelDel	8.5801
WALL.293	DelDel	8.5881
WALL.189	DelDel	8.6059
WALL.108	DelDel	8.6203
WALL.209	DelDel	8.6509
WALL.150	InsIns	8.6779
WALL.318	DelDel	8.6901
WALL.297	DelDel	8.7222
WALL.251	DelDel	8.7278
WALL.316	InsIns	8.7464
WALL.225	InsIns	8.7484
WALL.317	DelDel	8.7978
WALL.179	DelDel	8.8204
WALL.151	DelDel	8.8670
WALL.260	InsIns	8.8867
WALL.143	InsIns	8.9186
WALL.303	DelDel	8.9319
WALL.271	InsIns	8.9881
WALL.010	DelDel	9.0043
WALL.172	DelDel	9.0293
WALL.006	DelDel	9.1604
WALL.218	DelDel	9.2357
WALL.216	DelDel	9.3600
WALL.165	DelDel	9.4580
WALL.149	InsIns	9.7672

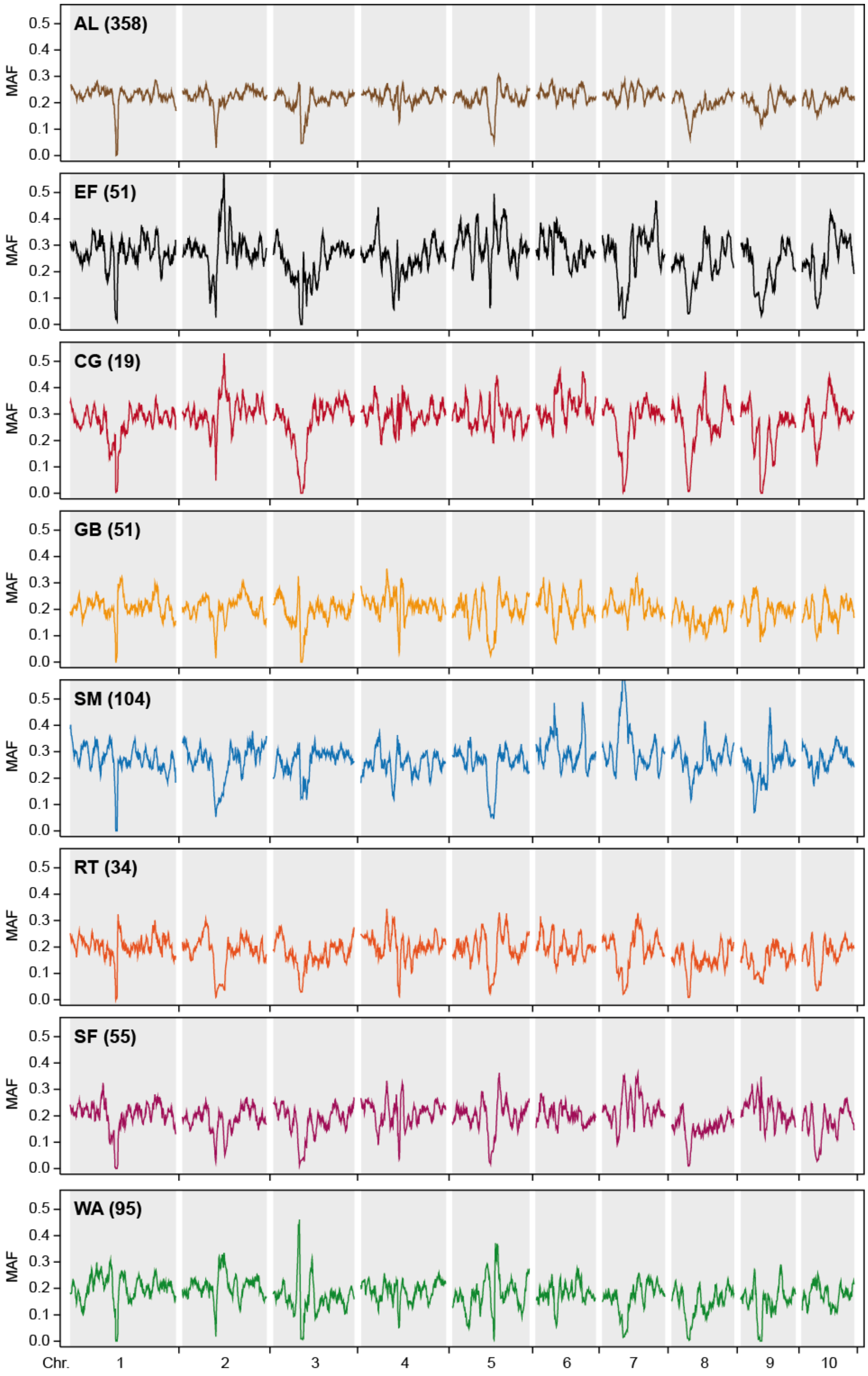

Fig. S1 Minor allele frequency (MAF). Frequency of the minor allele, assessed along the chromosomes by a sliding window approach, for the entire panel of 358 doubled haploid lines from all landraces (AL, as reference for determining the minor allele), the elite Flint (EF) lines, and in each of the six landraces.

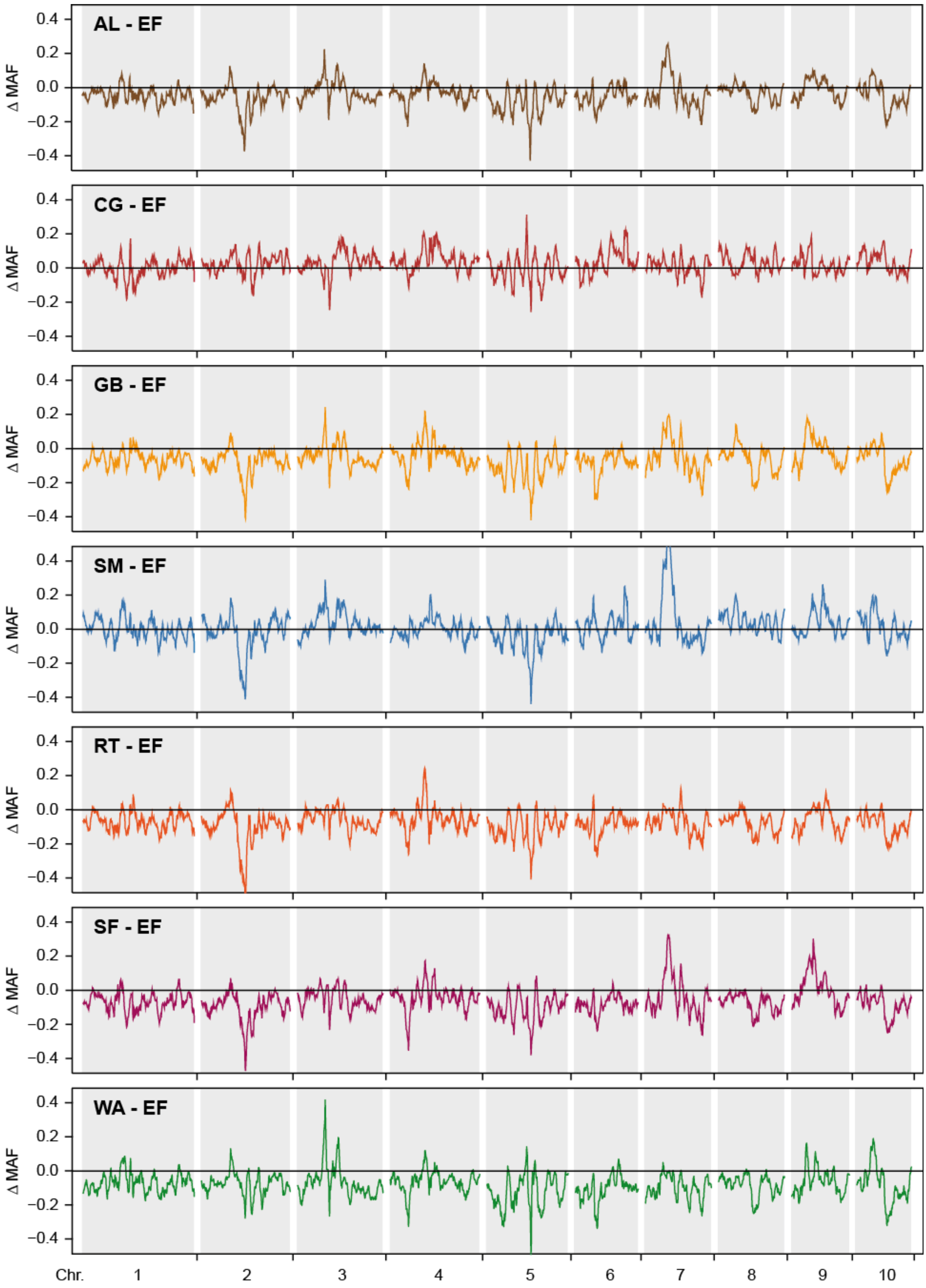

Fig. S2 Difference in minor allele frequency (Δ MAF) between ancestral landraces and elite Flint lines. Δ MAF assessed by a sliding window approach along chromosomes for the difference in MAF between the ancestral landraces (AL) and the elite Flint (EF) lines, and for each landrace and the elite lines.

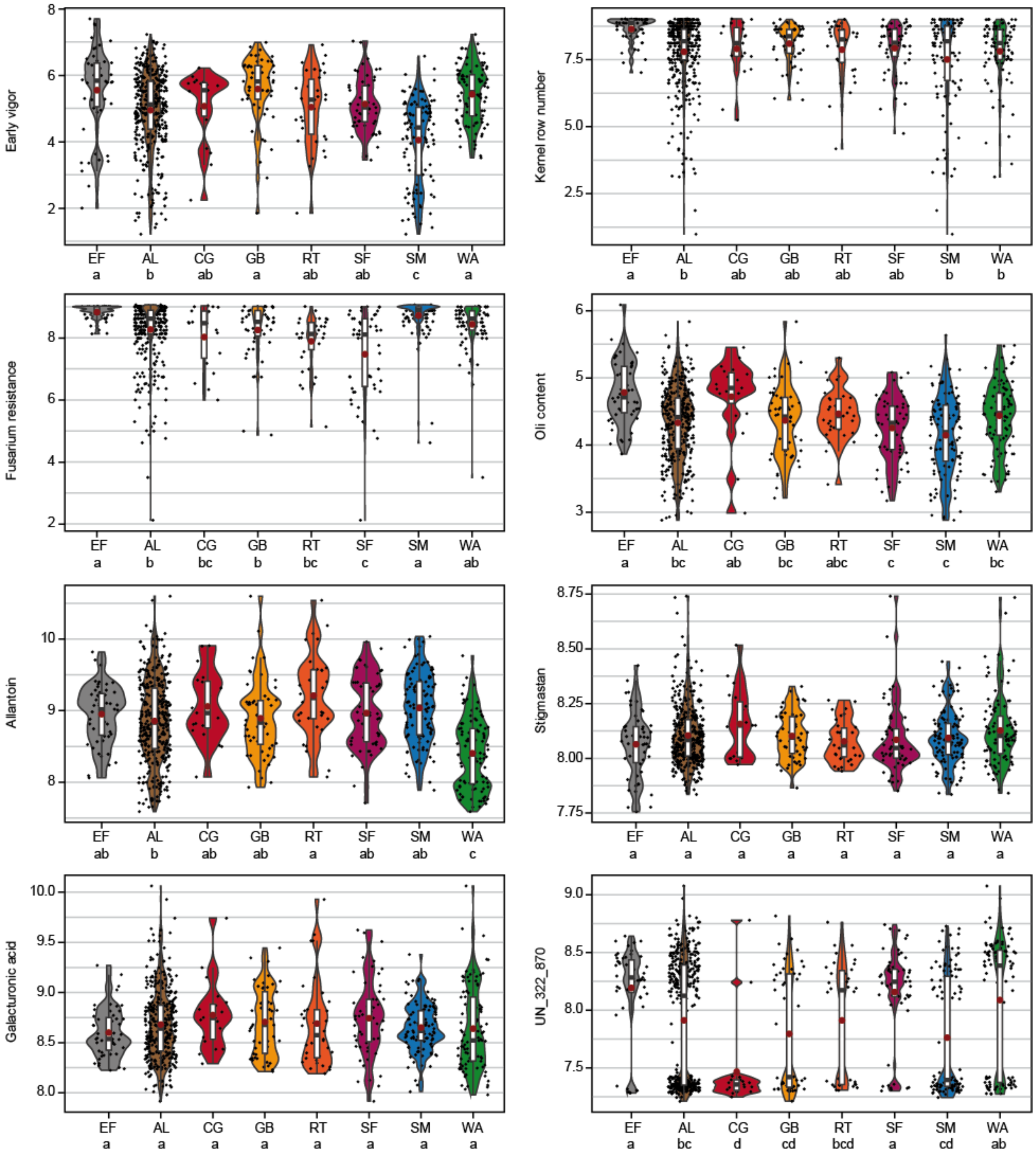

Fig. S3 Phenotypic variation in ancestral landraces and elite Flint lines. Violin plots showing the variation for the four agronomic traits and the four metabolites for 51 elite Flint (EF) lines, the entire panel of 358 doubled haploid lines from all landraces (AL) and the doubled haploid lines from each landrace. Letters indicate significant differences between the groups at the 0.05 significance level.

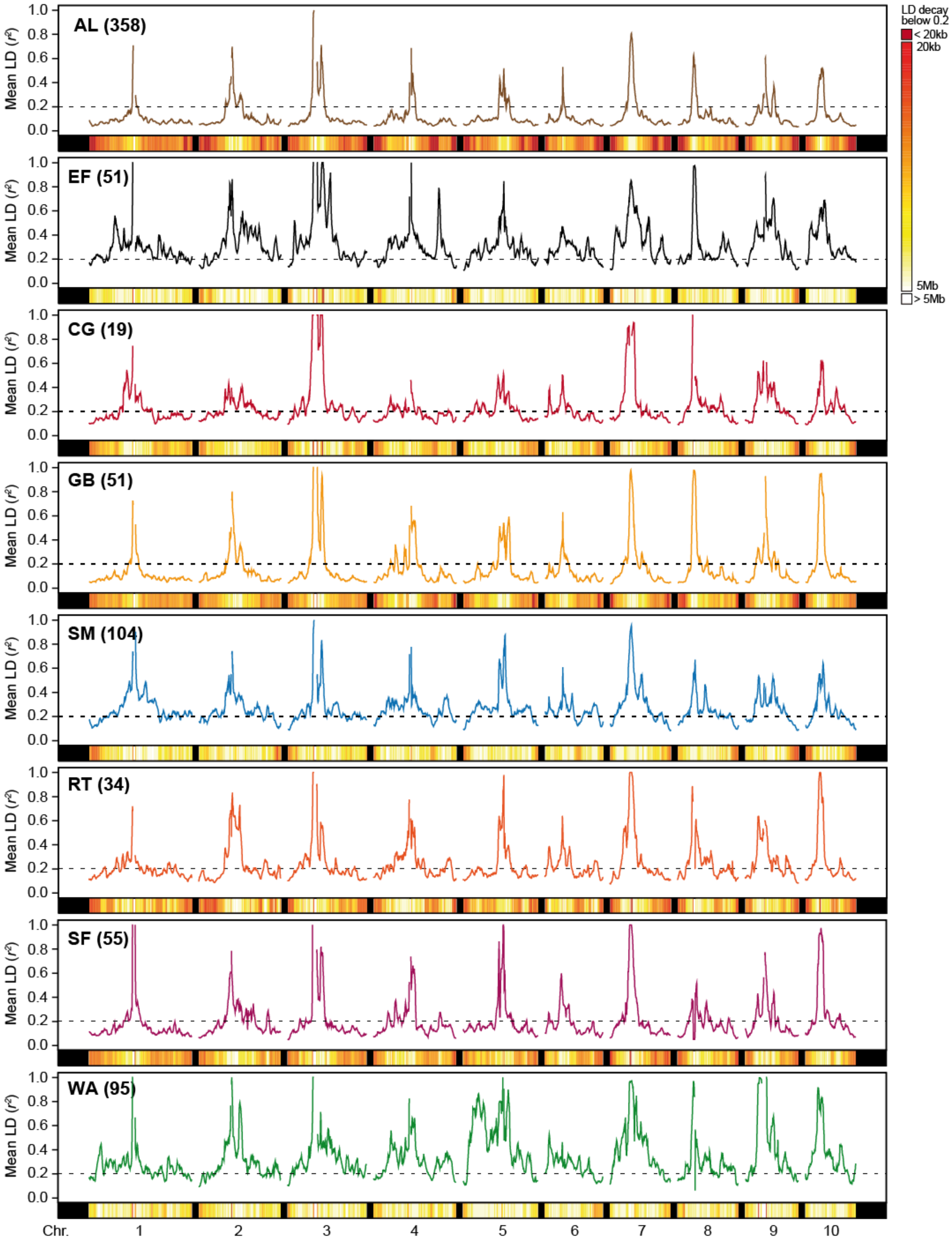

Fig. S4 Linkage disequilibrium (LD) along chromosomes. Mean LD assessed by a sliding window approach along chromosomes of the ancestral landraces (AL), the elite Flint (EF) lines and each of the six landraces. The heatmap bars underneath illustrate the physical distance after which the LD decays below 0.2.

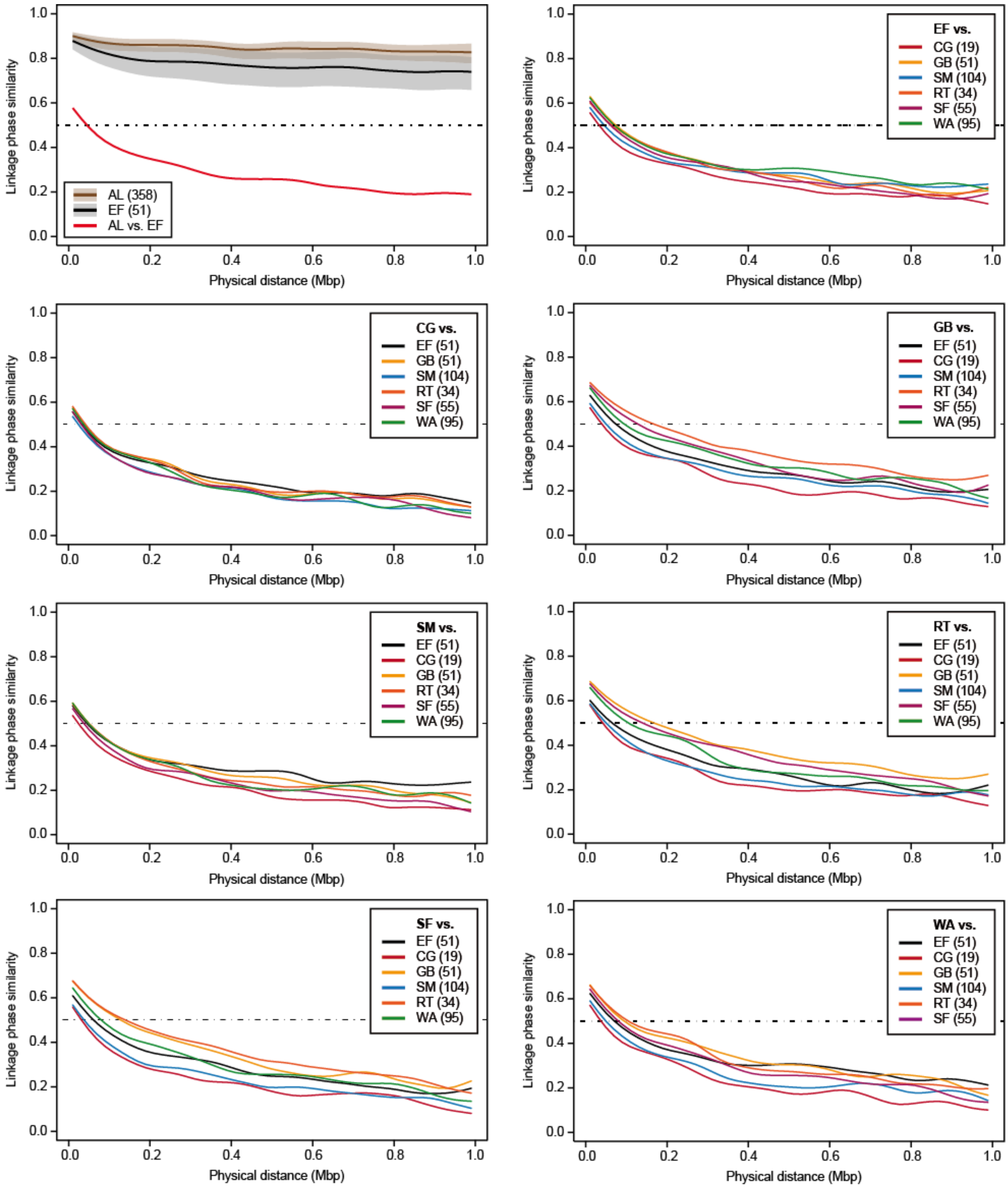

Fig. S5 Linkage phase similarity. Persistence of the same linkage phase between adjacent or close marker pairs within the ancestral landraces and within the elite Flint lines, as well as between these two groups, and between each landrace and all others.

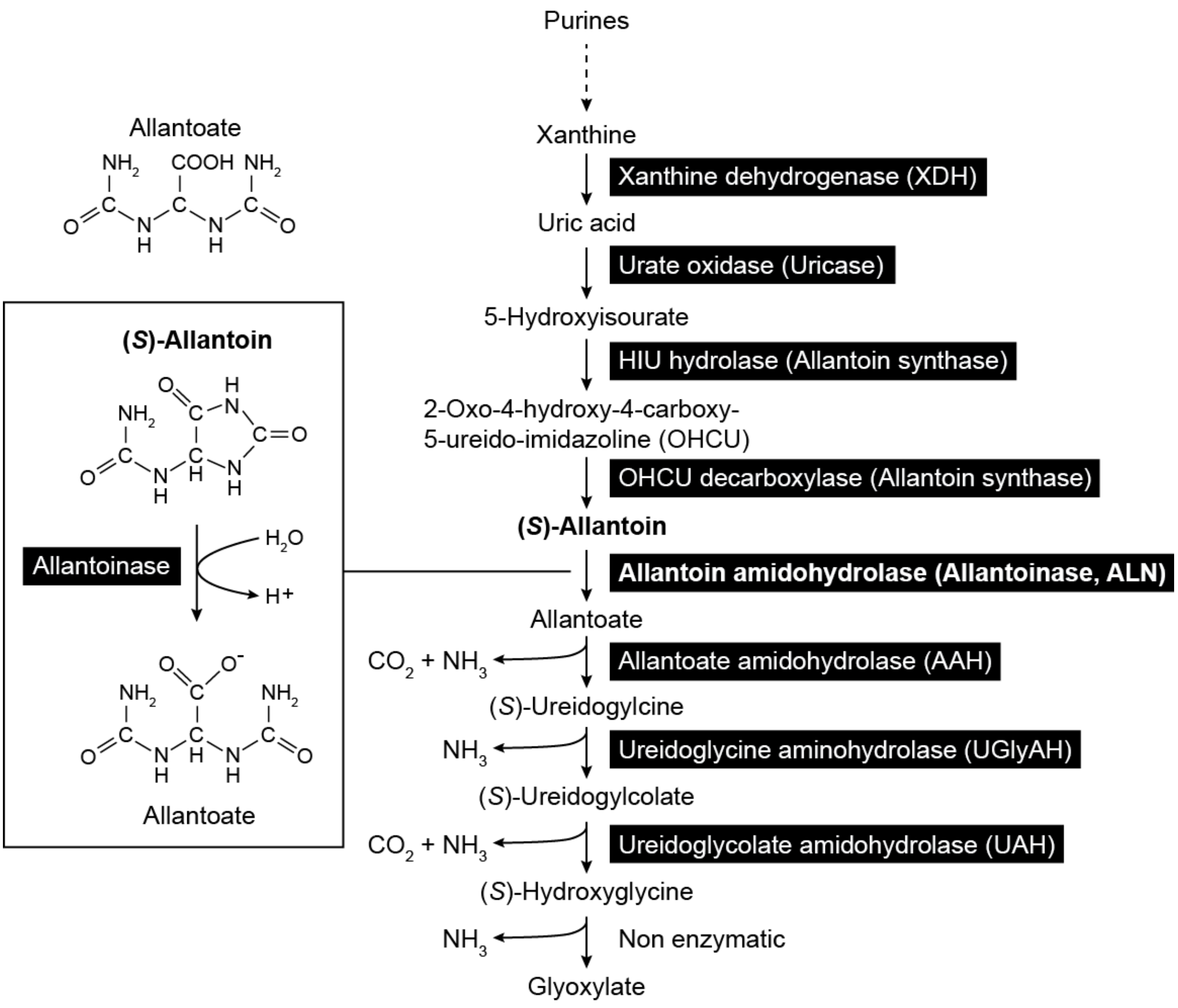

Fig. S6 Allantoin synthesis as part of the purine ring catabolism.

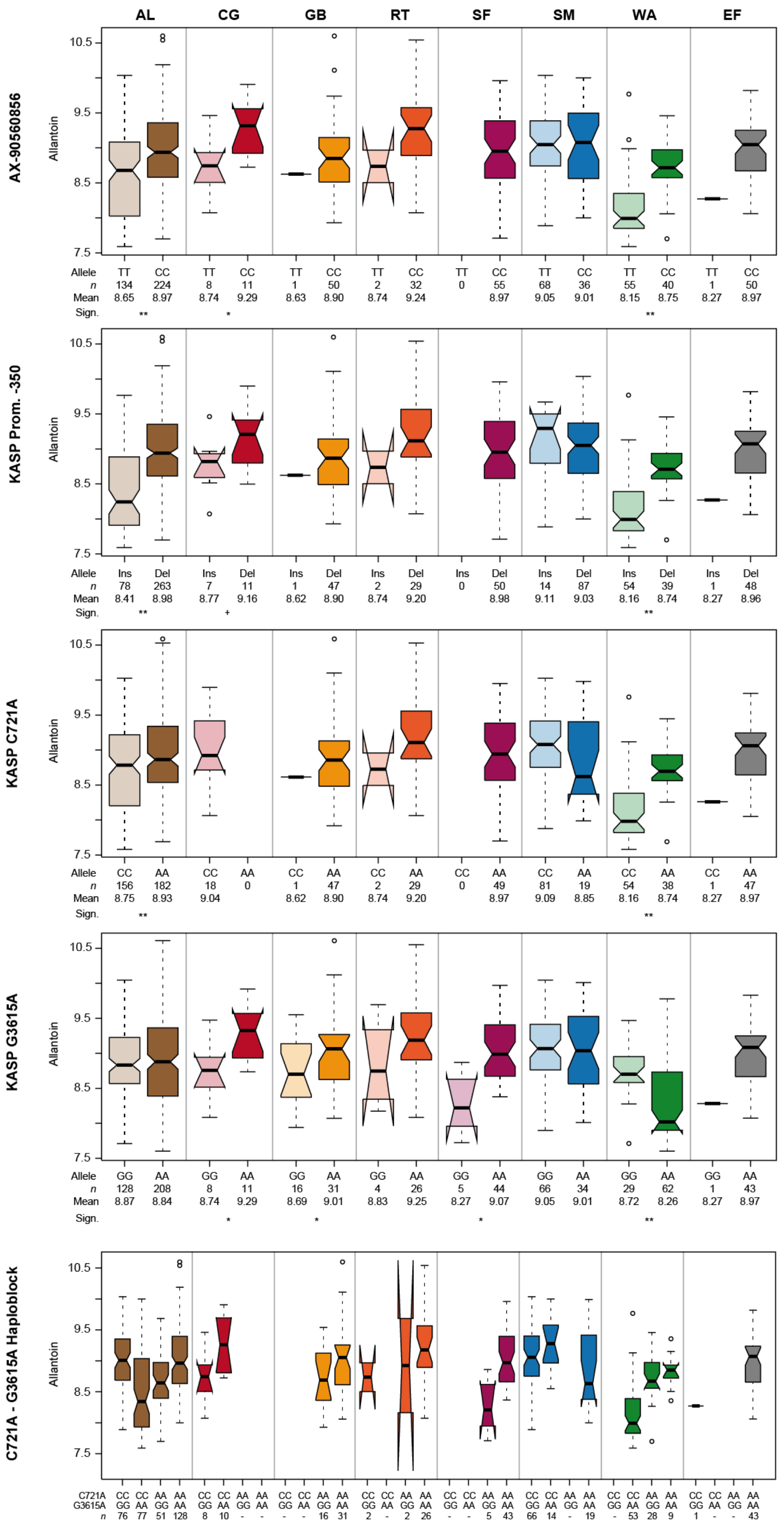

Fig. S7 Boxplots for allantoin content dependent on the allelic state at marker AX-90560856 and the KASP markers developed for identified polymorphisms. Marker AX-90560856 was identified in the genome-wide scan.

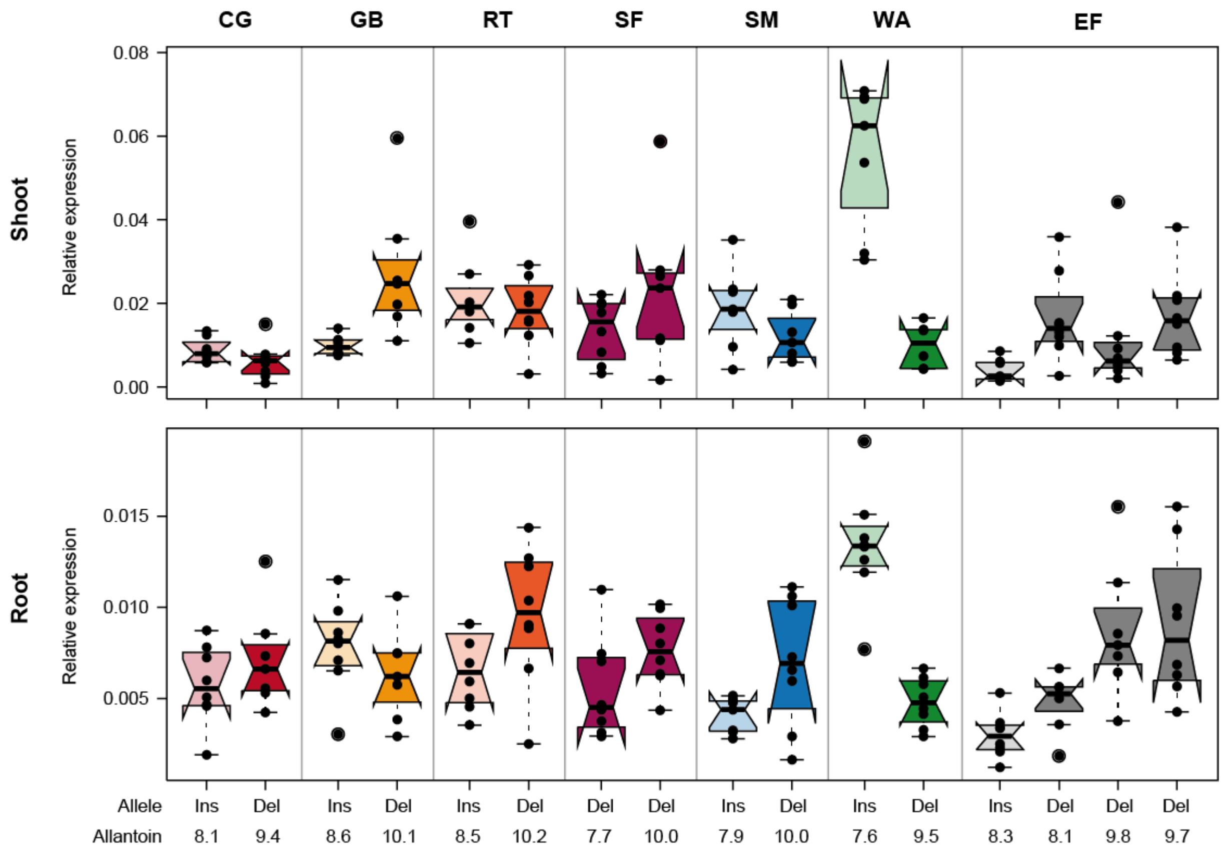

Fig. S8 Expression of *Allantoinase* mRNA. Boxplots showing the results from qPCR for *Allantoinase* expression in shoots and roots of maize seedlings in a discovery set of 16 lines with either the insertion (Ins) or the deletion (Del) in the promoter at position -350 bp. In addition to the allelic state, the allantoin content of each line is given.
